# Toxic Ag^+^ detection based on Au@Ag core shell nanostructure formation using Tannic acid assisted synthesis of Pullulan stabilized gold nanoparticles

**DOI:** 10.1038/s41598-023-27406-9

**Published:** 2023-02-01

**Authors:** Titilope John Jayeoye, Chamaiporn Supachettapun, Nongnuj Muangsin

**Affiliations:** 1 Department of Chemistry, Faculty of Science, Chulalongkorn University, Bangkok, 10330 Thailand; 2 Program in Petrochemistry and Polymer Science, Faculty of Science, Chulalongkorn University, Bangkok, 10330 Thailand; 3 Nanotec-CU Center of Excellence on Food and Agriculture, Department of Chemistry, Faculty of Science, Chulalongkorn University, Bangkok, 10330 Thailand

**Keywords:** Sensors, Chemistry, Materials science

## Abstract

Herein, a sensitive colorimetric detection strategy is proposed for Ag^+^ detection based on the use of environmentally friendly synthesis of gold nanoparticles (AuNPs), at room temperature, using (tannic acid, TA), as the reductant and pullulan (PUL) as stabilizing agent. The colloidal solution (TA/PUL-AuNPs), at the optimal synthesis conditions, showed maximum absorbance at 529 nm with a berry red color. TEM and FESEM validated that the particles are spherical and monodispersed, while other characterization results elucidated the role of pullulan in the nano-synthesis. Ag^+^ addition to the probe (TA/PUL-AuNPs), pH 11, resulted in naked-eye color changes, owing to Au@Ag core shell nanostructure formation. Further, the added Ag^+^ is reduced to AgNPs, on the surface of the TA/PUL-AuNPs probe. A hypsochromic shift in the absorption maximum, from 529 to 409 nm was observed, while (A_Ag+_-A_bl_)@409 nm exhibited linearity with Ag^+^ concentrations, from 0.100 to 150 µM. The estimated limit of detection was 30.8 nM, which is far lower than the acceptable limit of 0.930 µM from the regulatory agency. The TA/PUL-AuNPs probe was further tested for Ag^+^ detection in lake water samples, and it displayed satisfactory detection performances for real sample applications.

## Introduction

Human, health, and environmental pollutions arising from unguarded exploitation of resources is worrisome. When there is an imbalance in resource utilization, beyond nature´s capacity to replenish, there is bound to manifest, negative consequences. Heavy metals (HMs), pollution is quite prevalent, particularly of the common environmental matrices (air, water, and soil), owing to human-induced activities. HMs are metals with density greater than 5 kg/m^3^^1^. They are of major interest, by virtue of their toxicity to humans and animals. HMs of significant importance include Ag, Hg, Cd. Pb, As, Au, Cu and Ni. Among the listed HMs, keen interest is devoted to silver ion (Ag^+^), which may not be unconnected with the eco-physiological effects of Ag^+^ contamination^2^. Moreover, the incorporation of Ag^+^ into human consumer products due to its antibacterial property has consequently resulted in increased pollution of water bodies. In the human system, Ag^+^ can bind strongly with proteins, amino-acids, nucleic acids, owing to its sulfhydryl groups affinity, thereby forming complexes, which may induce deleterious effects on the optimal functioning of our body system. This can cause health damages on nerve cells, skin pigmentation and a weakened immune system^3,4^. As a result, the need for compassionate monitoring of Ag^+^ in the environment has continued to pique scientists’ interest. In fact, the maximum permissible limit of Ag^+^ in drinking water is set as 0.93 µM by the United State Environmental Protection Agency (USEPA)^5^.

Reported detection methods for Ag^+^ are, inductively coupled plasma-mass spectrometry (ICP-MS)^6^, atomic absorption/emission spectroscopy (AAS/AES)^7,8^. Electrochemical methods using carefully fabricated electrode^9,10^, detection assays based on fluorescence^11,12^. Others include, metal nanoparticles (MNPs) based colorimetric assays^13–15^. These methods are capable of reliable quantification of Ag^+^, however, some inherent challenges in the detection strategy call for a more efficient, facile, and environmentally benign method, with better detection performances. The use of environmentally friendly materials in nanomaterials fabrication, which can reduce some of their inherent toxicity profiles, has attracted huge attention in the past few decades^16,17^. This motivated us to use TA and PUL, materials with both plant and animal sources respectively, of undoubted environmental friendliness. Thus, the present synthesis strategy falls within the purview of the vigorously pushed green chemistry.

Biopolymers mediated fabrication of MNPs have continued to gain wide acceptance in the scientific community owing to their excellent water dispersity, abundance, non-toxicity, stability, biocompatibility, biodegradability, and eco-friendly properties. The presence of multitudinous hydroxy groups in their structures imbue them with reductive properties towards metal ion salts and thus have been well exploited for various MNPs fabrication^18–21^. Among the available biopolymers, pullulan has continued to generate keen interest. It’s perfect gel forming property in water, coupled with its ordered hydroxy groups have placed it in an advantaged position for multifarious application in comparison with other biopolymers^22^. Pullulan (C_6_H_10_O_5_)_n_, often produced from starch, through growing fungus (*Aureobasidium pullulans*), is composed of repeating maltotriose structures, with the glucose sub-units linked through an α-(1,4) glycosidic linkages, while the maltotriose units are linked by an α-(1,6) glycosidic bonds^23^. Pullulan is well suited for its diverse applications, because of its non-ionic, non-toxic, edible, biodegradable, and moderate viscosity properties^24^. These properties could be well extended into MNPs fabrication with a view to modulating the functional property of the synthesized materials. Thus, Laksee et al., synthesized stable and well dispersed AuNPs, using pullulan as the reductant, without any other reducing agent^25^. Owing to the poor reductive capacity of pullulan at ambient condition, the synthesis was operated at 80 °C, and was applied for the delivery of anticancer drugs. Further, the same group, synthesized pullulan stabilized AuNPs through prior modification of native pullulan, by quaternization step to impart positive charges on pullulan and further modification with para-aminobenzoic acid^26^. These two-steps modification processes of native pullulan, as explained, was for assisting in the delivery of anticancer drug (Doxorubicin), by the synthesized AuNPs, through ordered intermolecular interactions.

AuNPs as part of the plasmonic MNPs, have attracted considerable attention, due to its unique physico-chemical properties. AuNPs are biocompatible, easily tuned through diverse chemical reactions, exhibiting strong localized surface plasmon resonance (LSPR), that depends on the size, dielectric characteristics of the medium in which they are embedded and shape. Moreover, they show, high surface area to volume ratio, magnetic, optical and electronic property far exceeding other fluorophores and dyes^27^. In fact, AuNPs based colorimetric assays present advantages in terms of sensitivity, simplicity, reproducibility, and robustness. The detection systems can also be visualized by the naked eyes, while the reaction media can be read with the common UV–Vis spectrophotometer. Consequently, AuNPs-based detection have been applied to all kinds of analytes with comparable sensitivities, that can rival the popular/common detection methods^28–30^ etc.

In this contribution, a facile room temperature-based synthesis of PUL-AuNPs is reported, using common polyphenol (Tannic acid), as the reductant of gold salts, while the good aqueous solubility property of PUL was adopted to provide stability, for the fabricated colloidal nanocomposite. It was hypothesized, that while TA served as the reducing agent of gold salt, excess TA, may also be immobilized or fixed in the PUL matrix of the fabricated PUL-AuNPs and thus help in the in-situ reduction of Ag^+^ to AgNPs and thus present a detection strategy for Ag^+^ in aqueous environment. With the addition of Ag^+^ to the TA/PUL-AuNPs probe, coupled with pH adjustment, there are observable color perturbations from red, through orange and sparkling yellow, all within 6 min incubation at RT. The absorption maximum of TA/PUL-AuNPs did undergo a blue shift from 529 to 409 nm with increased intensity. The mechanism underlying the assay was elucidated and attributed to the generation of Au@Ag core shell nanostructure, where the fabricated TA/PUL-AuNPs acted as catalyst, to facilitate the in-situ reduction of Ag^+^ on the already-formed TA/PUL-AuNPs. The efficacy of the proposed assay in real sample Ag^+^ quantification in lake water sample, was equally demonstrated.

## Materials and methods

### Reagents

Pullulan with Mwt of 50–70 kDa was obtained from TCL Japan. Gold chloride trihydrate and Tannic acid were from Sigma Aldrich. EDTA was from BDH Industries Limited England. NaOH and AgNO_3_ 99.8% and were procured from RCI Labscan Thailand. Other metal salts used for selectivity studies include: Pb(NO_3_)_2_ and CdCl_2_.2.5H_2_O were from Sigma Aldrich, NaNO_3_ and KNO_3_ from Spectrum Chem Corp USA, FeSO_4_.7H_2_O was from Merck, MgCl_2_.6H_2_O and MnCl_2_.4H_2_O from QReC, ZnSO_4_.7H_2_O, CuSO_4_.5H_2_O, BaCl_2_, NiCl_2_.8H_2_O, Al(NO_3_)_2_.9H_2_O are all from APS Ajax Finechem Ltd., Ca(NO_3_)_2_.4H_2_O, CoCl_2_.6H_2_O, Cr(NO_3_)_3_, FeCl_3_, HgCl_2_ from LOBA CHEMIE, The salts were prepared by dissolving appropriate mass in ultra-pure water, obtained from a Millipore water purification system.

### Synthesis of TA/PUL-AuNPs

Firstly, 8% Pullulan solution was prepared by dissolving 8 g of pullulan in 100 mL of water, while being stirred at RT. The solution was further raised to 60 °C and was kept for 1 h. The source of heat was disconnected, while the solution was maintained under stirring. It was later stored at 4 °C, overnight to ensure complete dissolution of the polymer.

TA/PUL-AuNPs was fabricated as follow. Into a beaker, protected with aluminum foil, 40 mL of 2.5% PUL (optimized concentration of biopolymer), was measured under stirring, then 0.8 mL of Tannic acid 18 mM was added, then 1.2 mL of 0.1 M NaOH and finally 1 mL of gold (III) chloride trihydrate (78 mM) was rapidly added. The mixture was maintained at room temperature, under vigorous stirring for 1 h, then stored in an amber bottle at 4 °C before use.

### Colorimetric detection of Ag^+^ using TA/PUL-AuNPs probe

For a 5.0 mL glass tube, 90 µL of TA/PUL-AuNPs was pipetted, appropriate volume of ultra-pure water was added for dilution, then 130 µL of 0.05 M freshly prepared NaOH was injected, after which 50 µL of different preparations Ag^+^ solution was injected, so that the final concentrations of Ag^+^ ranged from 0.000 to 150 µM, for a reaction volume of 2.0 mL. The concentration of TA/PUL-AuNPs in the reaction system was calculated to be 0.096 nM, using extinction coefficient of gold nanoparticles with average particles size of 25 nm, given as 2.93 × 10^9^ M^−1^ cm^−1^^31^. The mixture was mixed using a table vortex and was kept to incubate at RT for 6 min, then the absorption spectra and photographic images were collected on an Agilent 8453 UV–Vis spectrophotometer and Samsung android phone respectively.

### Instruments

All absorption spectra measurements were collected from Agilent 8453 UV–Vis spectrophotometer, Samsung Android phone was used for photo images acquisition. Transmission electron microscope (TEM) was collected on a JOEL, JEM 2010 Japan, for particles images observation, field emission scanning electron microscope (FESEM), from Apreo, FEI, which was coupled to an energy dispersive X-ray spectroscopy (EDX) set-up (X Max 80, Oxford Instrument, UK), was used for microstructural observation of PUL and TA/PUL-AuNPs and the elemental composition respectively. X-ray diffractogram (XRD), was acquired with a XRD diffractometer (Empyrean) under the following features: collection degree (5°–70°) having a Cu Kα incident beam, with the following properties, scan speed of 70.2 s and wavelength of 0.154 nm. Raman spectra information was obtained from a Raman microscope spectrometer, using RAMANforce from Nanophoton, ATR-FTIR-Nicolet iS50 from Thermo-Fisher was used to reveal functional groups changes. The temperature response of the polymer and polymer impregnated with AuNPs were subjected to TGA and DTG analysis using a thermogravimetric analyzer, TGA 8000, from Perkin Elmer USA. A small quantity of the sample was exposed to continuous heating progressively from 50 to 1000 ◦C at 10 ◦ C/ min in nitrogen. X-ray photoelectron spectroscopy (XPS) was investigated using a Kratos AXIS Ultra DLD XPS Spectrometer (Shimadzu, Japan). The instrument conditions include a base pressure during analysis of 4.0 × 10^–7^ N/m^2^, analysis area of 700*300 µm, X-ray source of (Al K alpha) monochromator, operating condition (Emission 10 mA Anode HT 15 kV), electron emission angle at 45 °C, hemispherical analyzer positioned at 180 degrees. Charge neutralizer was used for electron balance and the obtained data was calibrated by the C 1*s* of carbon. Dynamic Light Scattering (DLS) and Zeta-potential, on Malvern ZETASIZER NANO. A well diluted sample was measured into the machine capillary cell and then placed in the sample holder, with the machine set for (n = 100) on each run, while running each sample in triplicates.

### Real sample application of TA/PUL-AuNPs

The practical application of the TA/PUL-AuNPs probe for Ag^+^ detection was investigated on lake water sample from the Chulalongkorn University, Thailand. The water sample was collected using a polypropylene bottle and was refrigerated at 4 °C in the laboratory before use. Before analysis, the water was prepared as follows. After removal from the refrigerator, the water sample was allowed to maintain RT and was later centrifuged at 6000 rpm for 20 min. It was then passed through a 0.22-micron syringe filter, for complete removal of particles. The prepared sample was submitted for Ag determination using ICP-OES, while the remaining portion was used on the TA/PUL-AuNPs probe. Sample standard addition method was applied, and the accuracy of the probe was expressed as percent recovery, using the equation,${\text{Recovery}} = ({\text{C}}_{{\text{a}}} {-}{\text{C}}_{{\text{b}}} )/{\text{C}}_{0} \times 100\%$ knowing that, C_a_ = concentration of spiked, C_b_ = concentration of unspiked sample and C_0_ is the concentration of added standard.

## Results and discussion

### Synthesis of TA/PUL-AuNPs

**Figure 1:**
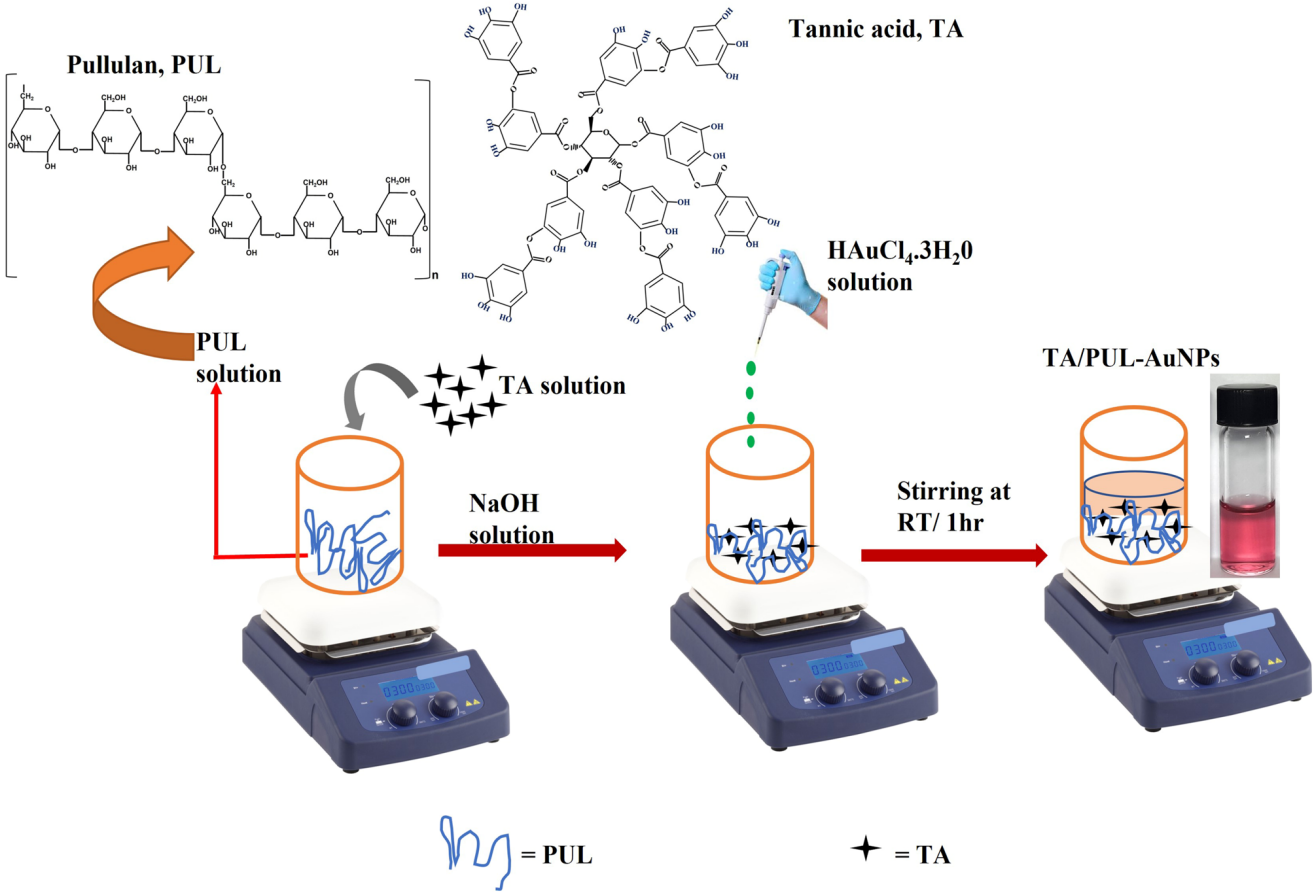
Schematics of synthesis protocol of TA/PUL-AuNPs at RT.

As detailed in Fig. 1, Tannic-a natural molecule with multi-hydroxyl functionality with huge biological properties was used as a reductant of gold salt inside pullulan matrix. Pullulan is a notable non-toxic and biocompatible biopolymer, whose aqueous solubility has been exploited in film forming applications for sustainable food packaging materials and in biomedicine^32^. Pullulan has multiple hydroxyl groups and thus can interact with TA, through *in-situ* hydrogen bonding formation, similar to a previous work^33^, where TA was immobilized in quaternized chitosan matrix, for hydrogel fabrication towards wound healing application. Since TA is a polyphenol, pH alteration to the basic environment would enhance the OH groups deprotonation, and thus improve its reductive properties. The addition of gold salt to the TA/PUL mixture under NaOH pH alteration, led to an immediate deep color formation, which was allowed to stir for 1 h at RT, to complete the general seed formation, nucleation/particles growth and maturation processes, characteristic of plasmonic nanoparticles formation stages^34^. The formed AuNPs is stabilized in PUL matrix, while excess TA could also interact with PUL, through hydrogen bonding and thus firmly immobilized. The synthesized TA/PUL-AuNPs after appropriate dilution showed UV–Vis absorption maximum at 529 nm, with a berry red color (Fig. 1).

Equation 1 can also depict the synthesis protocol more succinctly,

1 $${\text{Pullulan}} + {\text{TA}} + ({\text{NaOH}}\;{\text{solution}}) + {\text{Au}}^{3 + } \to [{\text{Pullulan - TA}}/{\text{Au}}^{0} ] + {\text{excess}}\;{\text{TA}}$$

The concentration of PUL in the reaction mixture was found to determine the optical properties of the TA/PUL-AuNPs colloidal solution and was immediately optimized.

**Figure 2:**
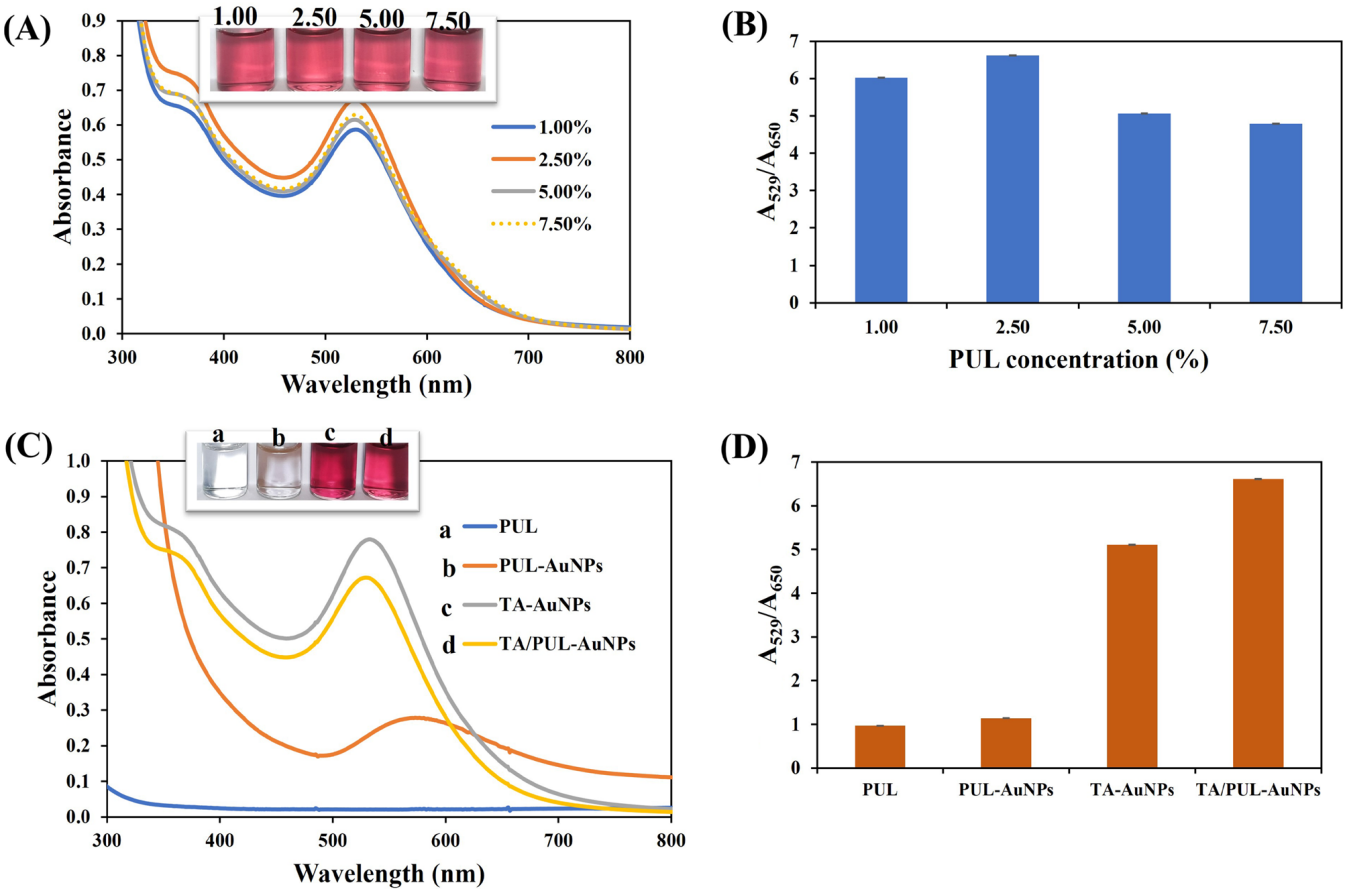
(**A**) UV–Vis absorption spectra of TA/PUL-AuNPs under different PUL concentrations (1.00–7.50%), inset shows the photo images. (**B**) Plot of A_529_/A_650_ against PUL concentrations. (**C**) UV–Vis absorption spectra of a. PUL, b. PUL-AuNPs, c. TA-AuNPs, d. TA/PUL-AuNPs, photo images are shown insets from a to d. (**D**) Plot of A_529_/A_650_ against the different synthesis materials.

As shown in Fig. 2A, the use of different concentrations of PUL does not impart significant changes on the absorption spectra nor, AuNPs color formed (Fig. 2A inset). Sharp absorption maximum at about 529–532 nm were obtained with berry red colors. However, further information on the dispersity of the colloidal solution can be obtained from the plot of A_529_/A_650_, as depicted in Fig. 2B. Accordingly, 2.50% PUL, at 529 nm, furnished the best aqueous dispersity and was thus selected. The stability of TA/PUL-AuNPs using 2.50% PUL was investigated and displayed in Fig. S1. As can be seen, the absorption spectra remained unchanged, even over 22 weeks, which attested to its high stability. This may be attributed to the PUL matrix offering excellent stabilizing effect on the colloidal nanocomposite. The synthesis materials were varied to profile the roles of each species, as presented in Fig. 2C. As seen, PUL solution does not show any UV–Vis peak (Fig. 2C curve a), also without any color (Fig. 2C a-inset) observation. The use of native PUL for AuNPs synthesis at RT, shows only slight absorption peak increment (Fig. 2C curve b), with a very faint color formation (Fig. 2C image b-inset). This material was not diluted at all, but the other solutions were diluted ten times. This shows that PUL is incapable of effecting significant gold salt reduction to its nanoparticles form at ambient conditions. This is typical of most biopolymers, where an increased temperature, through heating is often required to supplement their reductive efficacy of metal ion salts, during nanoparticles synthesis^25,26,35^. As a natural antioxidant agent, the use of TA alone, without the biopolymer furnished TA-AuNPs with absorption maximum at 534 nm (Fig. 2C curve c), with a deeper berry red color (Fig. 2C image c-inset). Quite impressively, the synthesis acquired under both TA and PUL mix yielded TA/PUL-AuNPs with UV maximum at 529 nm (Fig. 2C curve d) and light berry red color (Fig. 2C image d-inset), though having less intensity compared to TA-AuNPs. This observation shows that PUL, aside from serving as a stabilizing agent, also participated in the nanoparticle’s formation processes, by modulating the surface energy of reaction environment^36^. Moreover, the reduction in UV peak intensity may have resulted from the thick polymer layer on the particles surfaces, which can provide further stabilization spheres on the nanoparticles. The plot of A_529_/A_650_, of the synthesis materials is shown in Fig. 2D. Accordingly, TA/PUL-AuNPs showed the highest peak, which further accentuated the synergistic roles of TA and PUL in the formation of stable and dispersed TA/PUL-AuNPs colloidal solution.

### Characterization of TA/PUL-AuNPs

**Figure 3:**
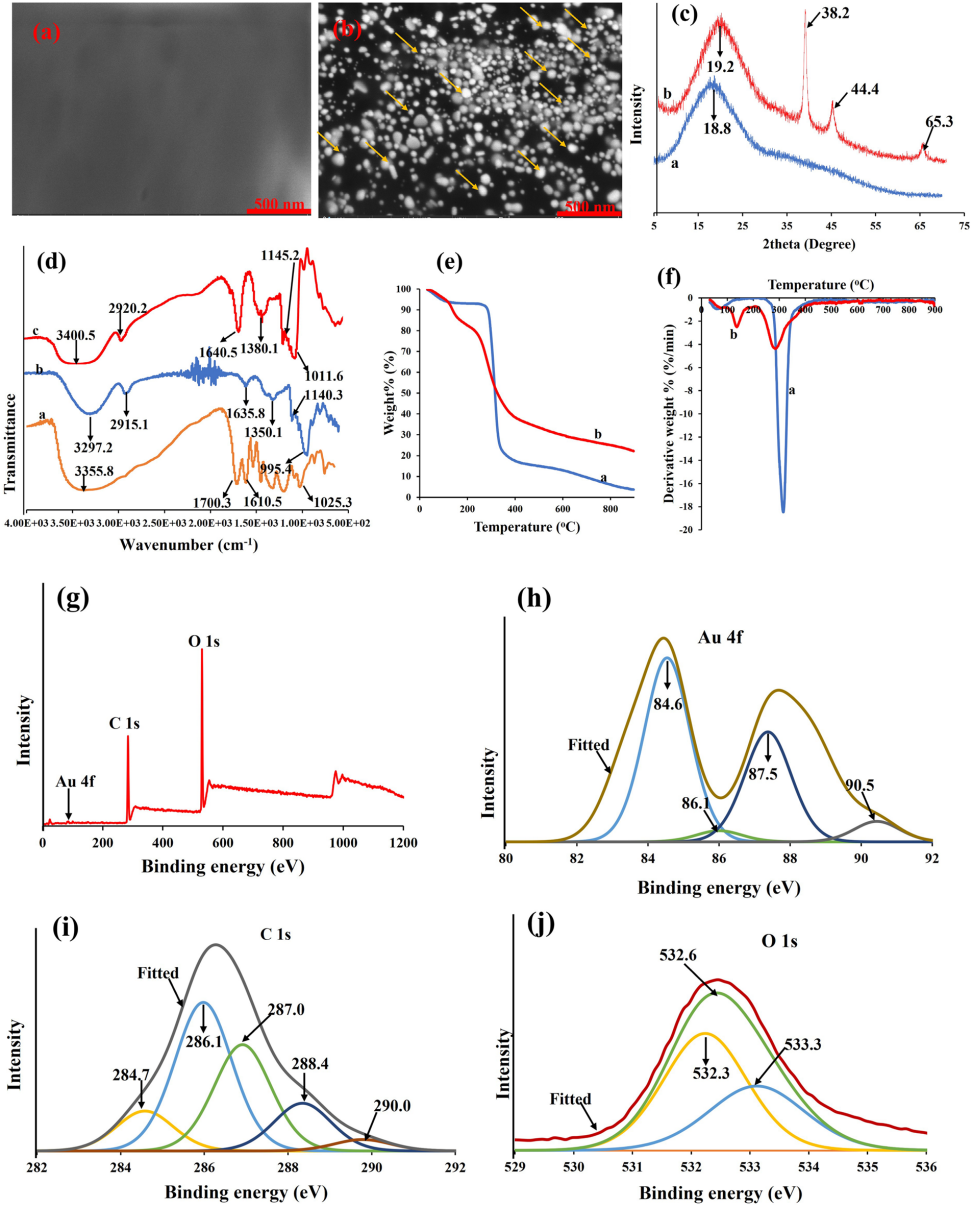
FESEM images of (**a**) PUL, (**b**) TA/PUL-AuNPs (AuNPs marked in yellow), (**c**) X-ray diffractogram of a. PUL, b. TA/PUL-AuNPs, (**d**) ATR-FTIR of a. TA, b. PUL, c. TA/PUL-AuNPs, (**e**) TGA (f) DTG of a. PUL, b. TA/PUL-AuNPs, (**g**) Full scan survey of TA/PUL-AuNPs, (**h**) binding energy of Au 4*f*, (**i**) C 1*s*, (**j**) O 1*s* deconvoluted spectra.

TA/PUL-AuNPs was subjected to detailed instrumental characterizations to reveal information on its the optical and microstructural properties. The TEM images of TA/PUL-AuNPs is shown in Fig. S2, revealing spherical and monodispersed particles. The particle size estimation of 50 particles from Image J Software is shown in Fig. S3, with major particles distribution around 20–30 nm. The hydrodynamic diameter estimated from a DLS machine shows particles sizes of about 66.5 ± 1.7 nm (Fig. S4), and zeta potential value of the as-synthesized TAPUL-AuNPs as − 15.4 ± 0.8 mV (Fig. S5). The increased particle size estimation, from DLS in comparison with TEM value arose from the differences in sample preparation. While DLS quantifies in aqueous solution and thus may estimate the hydration shell of the dispersant, in addition to the actual particle size^37^, TEM on the other hand is applied to dried sample. Moreover, the negative zeta value is reflective of the deprotonated pullulan carboxyl groups, which offers an effective stabilizing property to the nanoprobe. The FESEM images of PUL and TA/PUL-AuNPs is shown in Fig. 3a, b. The PUL film showed smooth surfaces (Fig. 3a), while the TA/PUL-AuNPs film showed rough surfaces, interspersed with numerous spherical particles (AuNPs), depicted with yellow arrows (Fig. 3b). This confirmed that the AuNPs are reduced inside the PUL matrix as stabilizing materials. Figure 3c, shows the X-ray diffractogram (XRD) of PUL and TA/PUL-AuNPs. Sharp peaks at 18.8° which is characteristic of Pullulan amorphous property is depicted (Fig. 3c, label a), while peaks at 19.2, 38.2, 44.4 and 65.3° are conspicuously revealed in TA/PUL-AuNPs (Fig. 3c, label b). These peaks belong to pullulan, while the (111), (200) and (311) is for the face centered cubic (*fcc*), AuNPs diffraction peaks. This shows that PUL plays a significant role in the optical property of the TA/PUL-AuNPs probe. The functional group interplay between TA, PUL and TA/PUL-AuNPs are investigated on ATR-FTIR and displayed in Fig. 3d. Identified peaks in TA, include—3355.8, 1700.3, 1610.5 and 1025.3 cm^−1^ (Fig. 3d, label a). These peaks signify the C-H stretching of the numerous hydroxyl groups in TA, the esters group functionality, the stretching peaks of the carboxylate ions COO^−^, and the C–O–C stretching vibration of the glycosidic linkages^38^. Peaks characteristic of PUL are found at, 3297.2, 2915.1, 1635.8, 1350.1, 1140.3 and 995.4 cm^−1^ (Fig. 3d, label b), which are reflective of the C-H stretching from O–H groups in pullulan, C-H stretching of aliphatic group, C–C stretching typical of aromatic groups. The peaks at 1140.3 and 995.4 cm^−1^ are typical of glycosidic linkages (α-1,4) and (α-1,6) of pullulan^39^. Figure 3d, label c), shows peaks of TA/PUL-AuNPs at, 3400.5, 2920.2, 1640.5, 1380.1, 1145.2 and 1011.6 cm^−1^, are close to the peaks identified in TA and PUL. This observation further confirmed the synergistic roles of TA and PUL, in directing the fabrication of TA/PUL-AuNPs probe. The Raman spectroscopy was used to further reveal the structural information and interaction between TA, PUL and TA/PUL-AuNPs, using the Surface Enhanced Raman Scattering (SERS) phenomenon, as shown in Fig. S6. Peaks at 1109.7, 1328.3, 1610.2 and 1705.9 cm^−1^ were identified in TA, correlated to the C-O stretching, O–H bending of phenol groups in TA, C = O and C = C stretching of multiple benzene ring of TA^40^. Identified peaks in PUL include-855.5 (C–O–C stretching), 930.8 (C–O–C asymmetric), 1132.7 (C–O–C asymmetric), 1350.6 (O–H bending vibrations), 1465.8 (C-H bending) and 2910.7 cm^−1^ (C-H stretching)^41^. Peaks in TA/PUL-AuNPs include-858.9, 1145.9, 1471.3, 1563.5 and 2905.9 cm^−1^, which are of diminished peaks in comparison with the TA and PUL peaks. This is a further confirmation for the synergistic influence of TA and PUL, in mediating the synthesis of the TA/PUL-AuNPs probe.

The thermal stability of PUL and TA/PUL-AuNPs were profiled on a TGA and DTG set up. The TGA profile (Fig. 3e), can be fitted into different degradation stages, as given in Table S1. For PUL (Fig. 3e, label a), three stages are identified. Stage 1 (25.30–250.4 °C), Stage II (250.5–500.1 °C) and Stage III (500.1–900.0 °C). The overall mass loss for the three stages is summed up at 96.5%, with a percent ash content of 3.50%. The three stages are assigned to the loss of moisture from the polymer, the degradation of the ordered polymer architecture and the complete carbonization stage of the polymer respectively. On the other hand, two major stages are assigned to the TA/PUL-AuNPs probe (Fig. 3e, label b). Stage I (25.40–250.4 °C) and Stage II (250.4–900.0 °C). The mass loss estimated was 77.8% with percent ash of 22.2%. The increased percent ash in the TA/PUL-AuNPs probe shows its increased thermal stability (over sixfold), compared to the polymer PUL. The derivative TGA is shown in Fig. 3f, which further revealed the identified degradation stages as temperature increased.

The X-ray photoelectron spectroscopy (XPS) was also used to profile the TA/PUL-AuNPs probe. The full survey of the material revealed peaks assigned to the Au 4*f*, C 1*s* and O 1*s* spectra (Fig. 3g). These peaks were de-convoluted and presented as high-resolution peaks of the identified elements. Figure 3h, shows the deconvoluted Au 4*f* spectra, with peaks at 84.6, 86.1, 87.5 and 90.5 eV. The peaks at (84.6 and 87.5 eV), are for Au 4*f*_7/2,_ Au 4*f*_5/2_ of Au^0^ (binding energy difference of about 3.0 eV) while the peaks (86.1 and 90.5 eV) are for Au 4*f*_7/2,_ Au 4*f*_5/2_ of Au^+^ respectively^42,43^. The small peak of Au^+^, shows that there existed some un-reduced Au salts, in the TA/PUL-AuNPs colloidal solution. In fact, the absorption maximum at 529 nm is a testament to that, as perfectly reduced AuNPs exhibit absorption maximum at 520 nm, with ruby red color. The C 1*s* high resolution spectrum is depicted in Fig. 3i, with peaks such as 284.7 (C–C, C–H), 286.1 (C–O), 287.0 (C–O–C), 288.4 (C=O) and 290.0 (O–C=O) eV^44^, all observed, corresponding to the different carbon chemical environment in the material. The O 1*s* spectrum is shown in Fig. 3j, with peaks at 532.3 (O–C=O), 532.6 (C–OH) and 533.3 (C–O) eV^44^, confirming oxygen atom linked to carbon atom in multi-dimensional conformations. These shows there existed strong interactions between AuNPs, TA and PUL, to create a stable, ordered network of colloidal nanocomposite of TA/PUL-AuNPs.

### TA/PUL-AuNPs colorimetric nanoprobe for Ag^+^ detection

**Figure 4:**
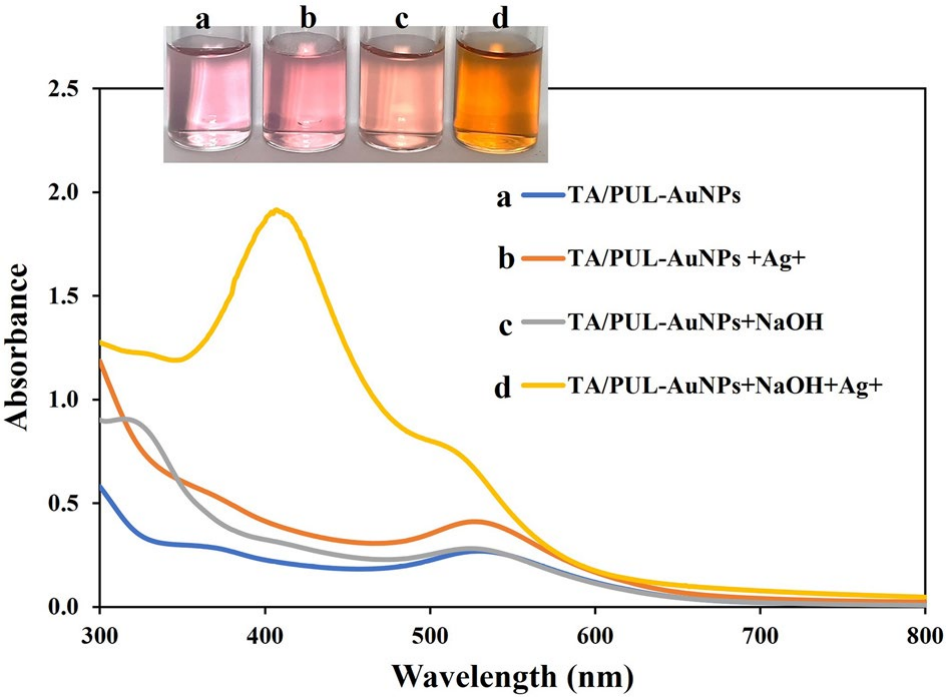
UV–Vis spectra of a. TA/PUL-AuNPs, b. TA/PUL-AuNPs with Ag^+^ addition, c. TA/PUL-AuNPs with NaOH addition, d. TA/PUL-AuNPs with NaOH and Ag^+^ addition, inset shows the photo images of a–d (Ag^+^ concentration = 150 µM, NaOH = 3.25 mM).

The detection capacity of TA/PUL-AuNPs for Ag^+^ in solution was firstly investigated by mere addition of the analyte to the colorimetric probe, without and with pH adjustment using NaOH solution. As shown in Fig. 4, TA/PUL-AuNPs show UV maximum at 529 nm (Fig. 4, curve a), with a faint berry red color (Fig. 4, image a-inset). The addition of Ag^+^ concentration at 150 µM (final concentration), imparted only a slight absorption maximum change (Fig. 4, curve b), with a red color (Fig. 4, image b-inset). NaOH mediated pH adjustment in TA/PUL-AuNPs do not impart a noticeable change in the absorption spectra (Fig. 4, curve c), but a slight change in color (Fig. 4, image c-inset) and another peak at 320 nm, which may be attributed to the activation of TA initially immanent in PUL Matrix^45^. The pH after NaOH based adjusted was at 11. This observation may be attributed to further activation of TA in the PUL matrix. The addition of Ag^+^ to a pH adjusted TA/PUL-AuNPs resulted in a hypsochromic shift in the absorption spectra, with an intensity enhancement (Fig. 4, curve d), while a rapid color change to sparkling yellow (Fig. 4, image d-inset), was observed. This shows that pH adjustment is sacrosanct for effective response to Ag^+^ using the proposed TA/PUL-AuNPs probe.

### Analytical features of TA/PUL-AuNPs in Ag^+^ detection

To achieve optimal detection response using TA/PUL-AuNPs probe, relevant parameters affecting the detection sensitivity must be appropriately optimized. In this light, the concentration of NaOH in the reaction system and the incubation time were optimized.

### Optimization of detection response

The effect of concentration of NaOH in the reaction system was optimized under 80.0 µM of Ag^+^, by varying the volumes of 50 mM freshly prepared NaOH solution used. 30, 60, 90, 130 and 150 µL of 50 mM was injected in the 2 mL, reaction pot so that the final concentrations of NaOH are (0.750, 1.50, 2.25, 3.25, 3.75 mM), respectively. Figure S7, shows the plot of NaOH concentration against the response. As shown, the response increased as the concentrations of NaOH increased, while showing only a slight response change between 3.25 and 3.75 mM. It was noted that 3.75 mM NaOH, could impact on the stability of the colloidal Au@Au core shell nanostructured formed over time^46^, consequently, NaOH of final concentration 3.25 mM, was thus selected for this work.

The incubation time was investigated by collecting the absorption spectra under different concentrations of Ag^+^ (2.50, 20.0, 80.0 and 150 µM), at 2 min interval, for 14 min. As revealed in Fig. S8, TA/PUL-AuNPs show rapid response with a saturation at about 6 min and were thus selected.

### Sensitivity

**Figure 5:**
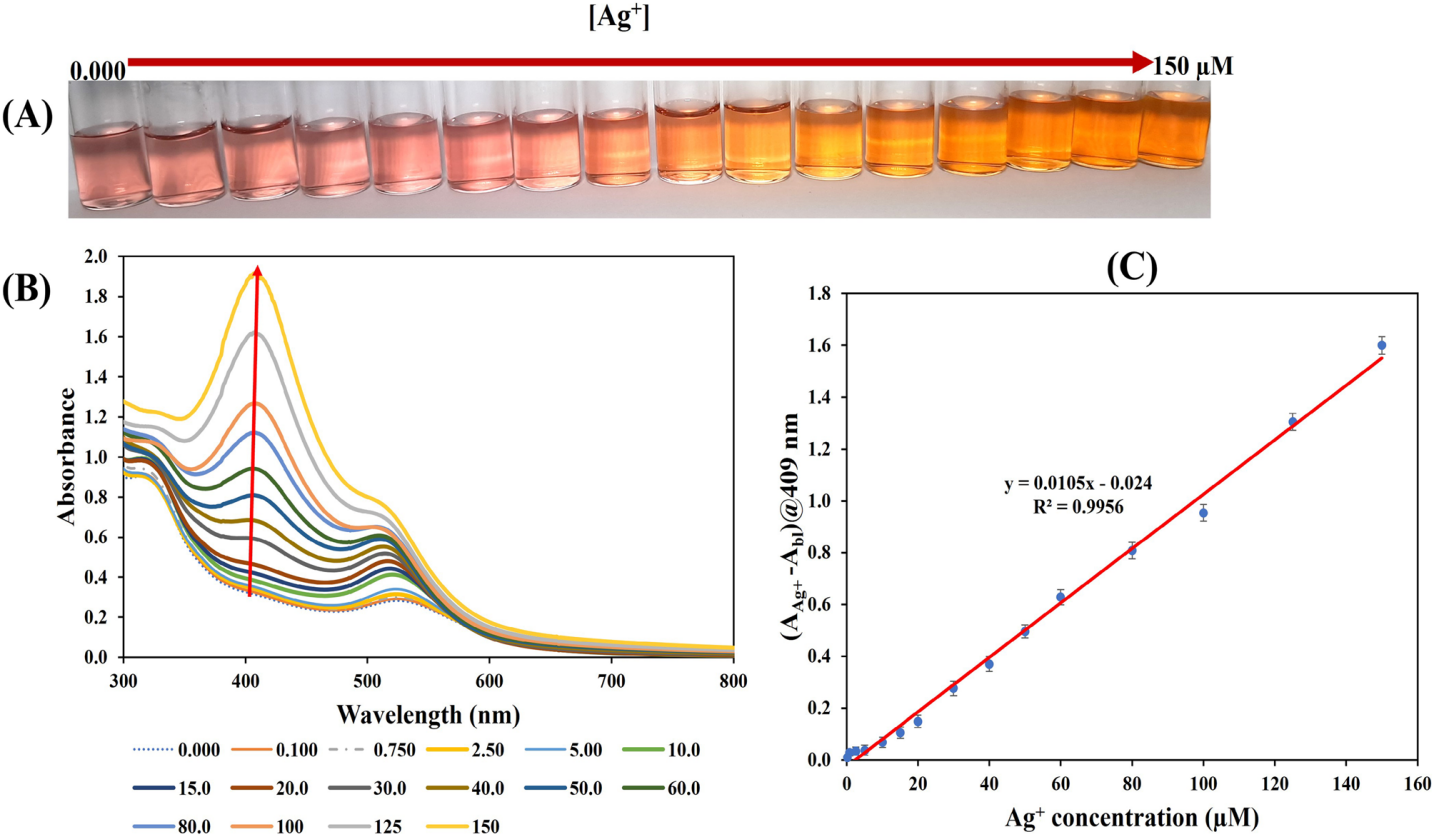
(**A**) Photo images of TA/PUL-AuNPs under different concentrations of Ag^+^ (0.000–150 µM), (**B**) UV–Vis spectra, (**C**) plot of Ag^+^ concentration versus detection response (A_Ag+_ − A_bl_)@409 nm, showing linearity between 0.100 and 150 µM.

At the optimum detection conditions, the sensitivity of the TA/PUL-AuNPs probe towards Ag^+^ detection in aqueous environment was investigated through charging different concentrations of Ag^+^, so that the final concentrations ranged from 0.000 to 150 µM. The addition of Ag^+^ to pH adjusted TA/PUL-AuNPs, vortex mixed and incubated at RT for 6 min resulted in naked-eye observable color changes (Fig. 5A). The color transitions ranges from red to faint yellow, yellow, and orange, which confirmed the efficacy of TA/PUL-AuNPs towards Ag^+^ in solution. Figure 5B, shows the UV–Vis spectra of TA/PUL-AuNPs under Ag^+^ injection, at the optimum detection conditions. As noted, the absorption spectra progressively blue-shifted (hypsochromic effect), with an increased intensity. As the concentrations of Ag^+^ increases, a sharp peak developed at around 409 nm which is typical of small-sized plasmonic AgNPs peak^47^. The UV–Vis spectra at the low concentrations (0.000–5.00) µM, is displayed in Fig. S9, to reveal the spectra shift more conspicuously at the low range. The analytical response selected for this work (A_Ag+_ − A_bl_)@409 nm, where A_Ag+_ is the absorbance intensity of the Ag^+^ analyte, A_bl_ is the absorbance intensity of the blank, both at 409 nm. The plot of Ag^+^ concentrations against (A_Ag+_ − A_bl_)@409 nm, shows linearity between 0.100–150 µM, Fig. 5C. The plot is presented in the form of equation, (A_Ag+ _− A_bl_)@409 nm = 0.0105 [Ag^+^] − 0.024, showing an R^2^ value of 0.9956. The limit of detection (LOD) was evaluated using the equation, 3.3xSy/x/slope, where Sy/x, (standard deviation of the regression), and the slope is obtained from the calibration curve (ICH, 1996)^48^. The LOD calculated 30.8 nM, is far below the acceptable level of Ag^+^ in drinking water, fixed at 0.930 µM from the United State Environmental Protection Agency (USEPA)^49^. Consequently, this sensor can determine Ag^+^ at environmentally relevant range, for concerned pollution monitoring.

### Proposed mechanistic basis of detection

**Figure 6:**
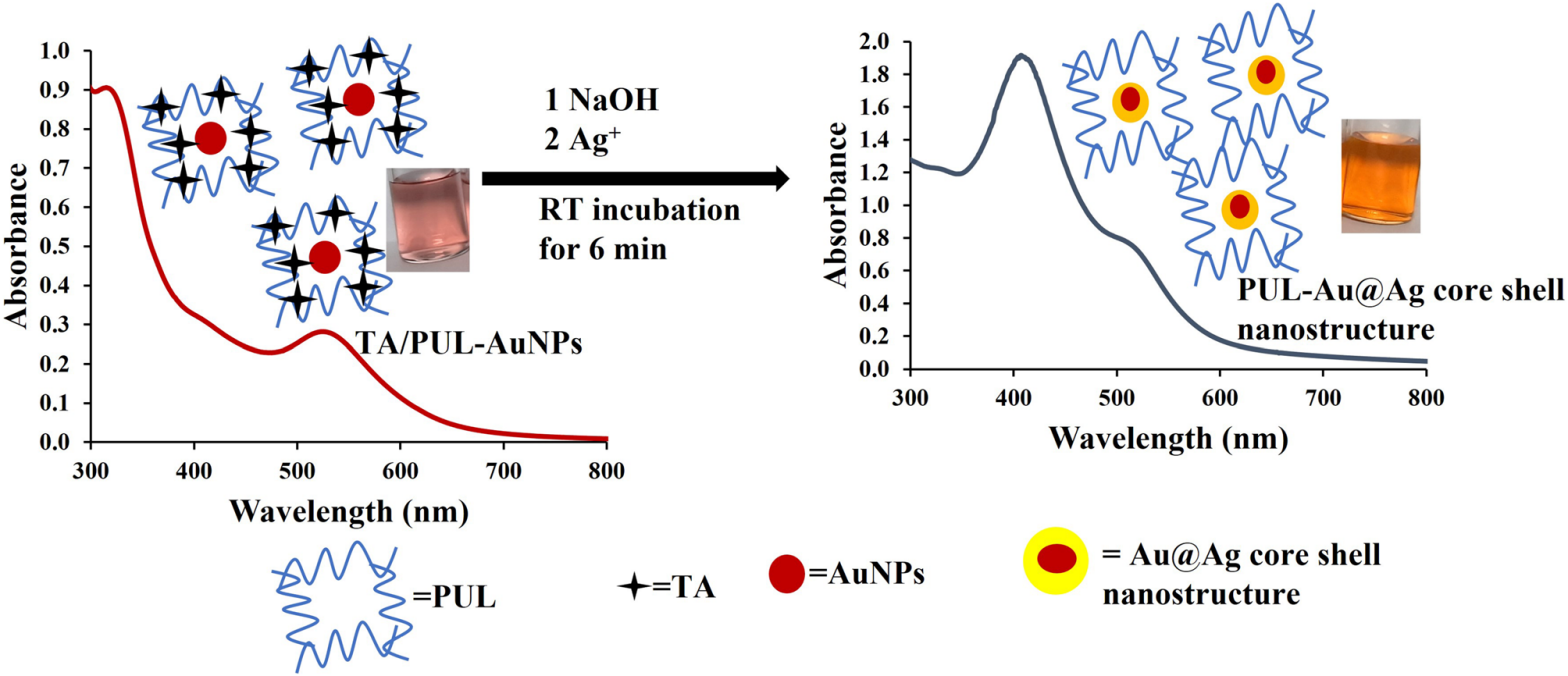
Mechanistic illustration of TAPUL-AuNPs based probe for Ag^+^ detection.

**Figure 7:**
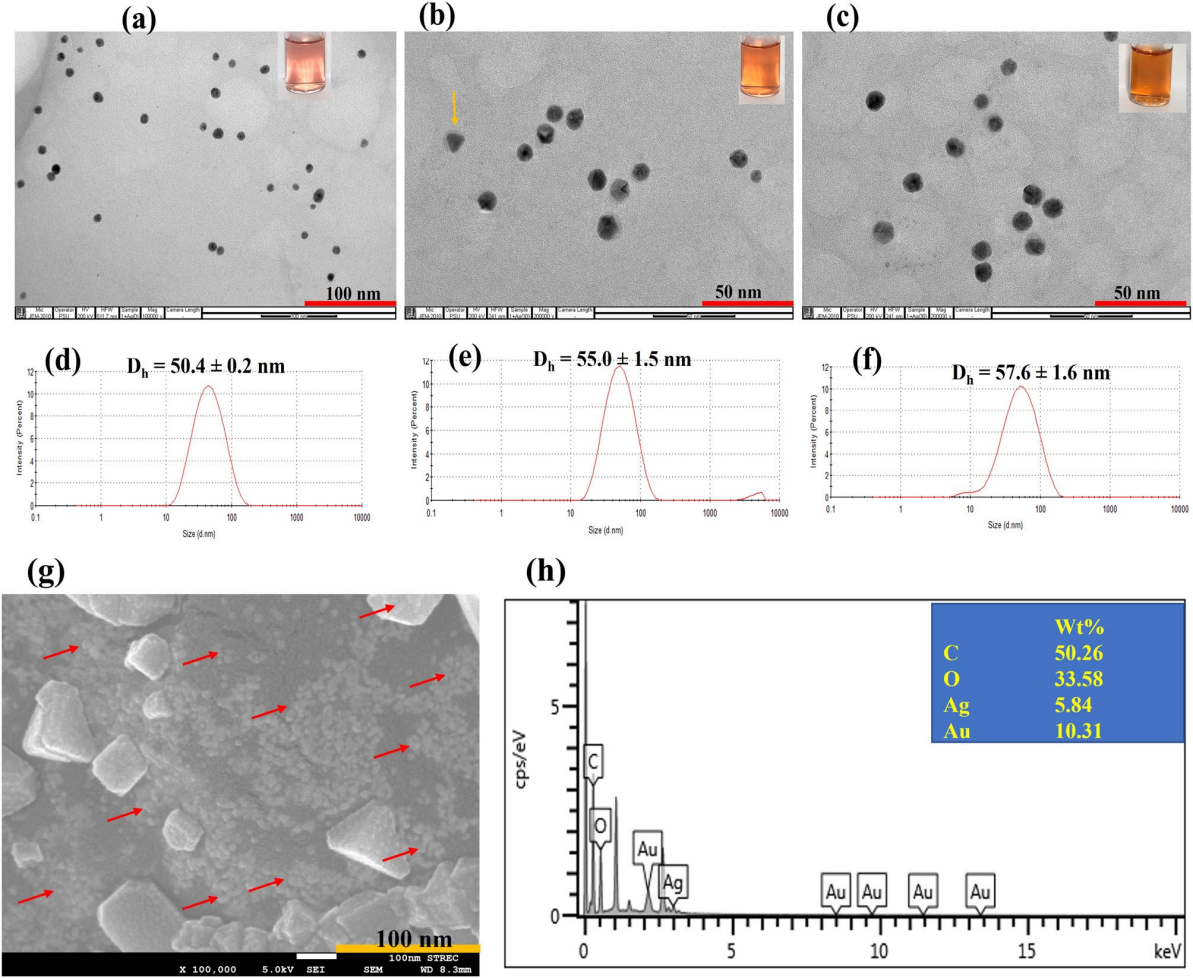
TEM images of pH adjusted TA/PUL-AuNPs with the addition of (**a**) 0.000, (**b**) 30.0, (**c**) 100 µM Ag^+^ (inset shows the photo images), while (**d**–**f**) represent the hydrodynamic diameters from DLS, corresponding to (**a**–**c**), (**g**) FESEM images of TA/PUL-AuNPs under 60.0 µM Ag^+^ injection (red arrows show the Au@Ag core shell nanostructure inside PUL matrix at high magnification), (**h**) EDS spectrum of TA/PUL-AuNPs under 60.0 µM Ag^+^ injection, showing Au and Ag peaks.

The possible mechanism of TA/PUL-AuNPs is explained as follows. From the exhaustive instrumental characterizations deployed to get detailed insight into the TA/PUL-AuNPs morphological and microstructural properties, it is right to conclude that TA, acted as the reductant of gold salt, all inside PUL matrix. While some TA were used up for gold salt reduction, to form AuNPs, excess TA are fixed or immobilized in the PUL Matrix through intermolecular hydrogen bonding. This intermolecular interaction is between the multitudinous OH groups in TA and PUL structures, as shown in Fig. 6. With NaOH addition, the excess TA would become activated and can thus facilitate *in-situ* reduction of Ag^+^, inside PUL matrix, when added to furnish new AgNPs, which is deposited on the initial AuNPs. Consequently, Au@Ag core shell nanoparticles is formed inside PUL matrix as (PUL-Au@Ag core shell nanostructure). Furthermore, the TA/PUL-AuNPs probe can act as a catalyst for the nucleation/growth steps, during *in-situ* AgNPs formation, and can thus be concluded within a short time frame. As a result, the TA/PUL-AuNPs can decrease the redox potential of the Ag^+^/Ag^0^ significantly^50^. It is also important to state that the similarity in the lattice parameters of Ag (0.4086 nm), and Au (0.4079 nm)^51^, also encouraged the swift formation of AgNPs on TA/PUL-AuNPs surfaces as observed, which is similar to the conventional seed mediated growth of nanoparticles formation^52^. This observation is marked with visible color perturbations and absorption spectra enhancement and a hypsochromic shift. The TEM images and the hydrodynamic diameter from DLS of TA/PUL-AuNPs under different Ag^+^ concentrations were acquired. As shown in Fig. 7, the TEM image of pH adjusted TA/PUL-AuNPs shows spherical particles (Fig. 7a), with reddish color (Fig. 7a, image-inset) and particle diameter of 50.4 ± 0.2 nm from DLS (Fig. 7d). However, on Ag^+^ injection at 30.0 µM, the TEM images show particles are more uniform in size, while some non-spherical images could be identified (as shown with yellow color arrow) (Fig. 7b). The color change to orange could be clearly noticed (Fig. 7b image-inset). The hydrodynamic diameter equally increased to 55.0 ± 1.5 nm (Fig. 6e). Furthermore on 100.0 µM injection of Ag^+^ on pH modulated TA/PUL-AuNPs, the TEM images are more uniformly distributed, with all spherical shapes (Fig. 7c). The color image is deep yellow (Fig. 7c, image-inset) and the DLS diameter was 57.6 ± 1.6 nm (Fig. 7f). The zeta potential of TA/PUL-AuNPs (detection conditions) with Ag^+^ treatment at (0.000, 30.0 and 100 µM), is shown in Fig. S10. As shown, there was a progressive increment in zeta value from − 16.0 ± 1.1 mV (Blank or 0.000 µM Ag^+^), to − 19.3 ± 2.2 mV for 30.0 µM Ag^+^ and − 21.6 ± 1.4 mV for 100 µM Ag^+^ injection. This trend shows the particles become more stable as more of the TA molecules were used for in-situ reduction of Ag^+^ to AgNPs, which further corroborated the trend obtained from TEM images observation. We further profiled the FESEM image and EDS spectrum of pH adjusted TA/PUL-AuNPs after 60.0 µM Ag^+^ addition, as displayed in Fig. 7g. The presence of spherical Au@Ag nanostructures (red arrows) is visibly distributed in the PUL polymer matrix (Fig. 7g) observed at high magnifications. The EDS spectrum (Fig. 7h) confirmed the presence of C, O, Au and Ag with varying percent distributions (Fig. 7h, inset). The C and O peaks, which are clearly from the biopolymer, while Au and Ag peaks, confirmed the generation of Au@Ag core shell nanostructure arising from the reduction of Ag^+^ on pre-formed TA/PUL-AuNPs. The elemental mapping (Fig. S11 further confirmed the presence of C, O, Au, and Ag, validating the result obtained from the EDS spectrum analysis.

### Precision

The precision of the TA/PUL-AuNPs probe for Ag^+^ detection was investigated through charging Ag^+^ concentrations at 30.0 and 100 µM, by acquiring eight replicates (n = 8) running on a single day for intra-day precision and same concentrations was acquired from three consecutive days, for inter-day precision. The precision was represented as relative standard deviation (RSD%). The estimated intra and inter-day precision are given as 0.8 and 2.6% respectively. This shows the detection strategy is very reliable and can detect Ag^+^ without significant response variability. The exceptional ability of biopolymers matrix to hold and deliver active materials is key to their indispensable applications in wide-ranging fields of human endeavors. Their functional groups can interact with different and thus confer enormous stability over time^51^. Thus, the PUL matrix can hold TA firmly in place, which enhances the detection robustness of the proposed assay.

### Selectivity

**Figure 8:**
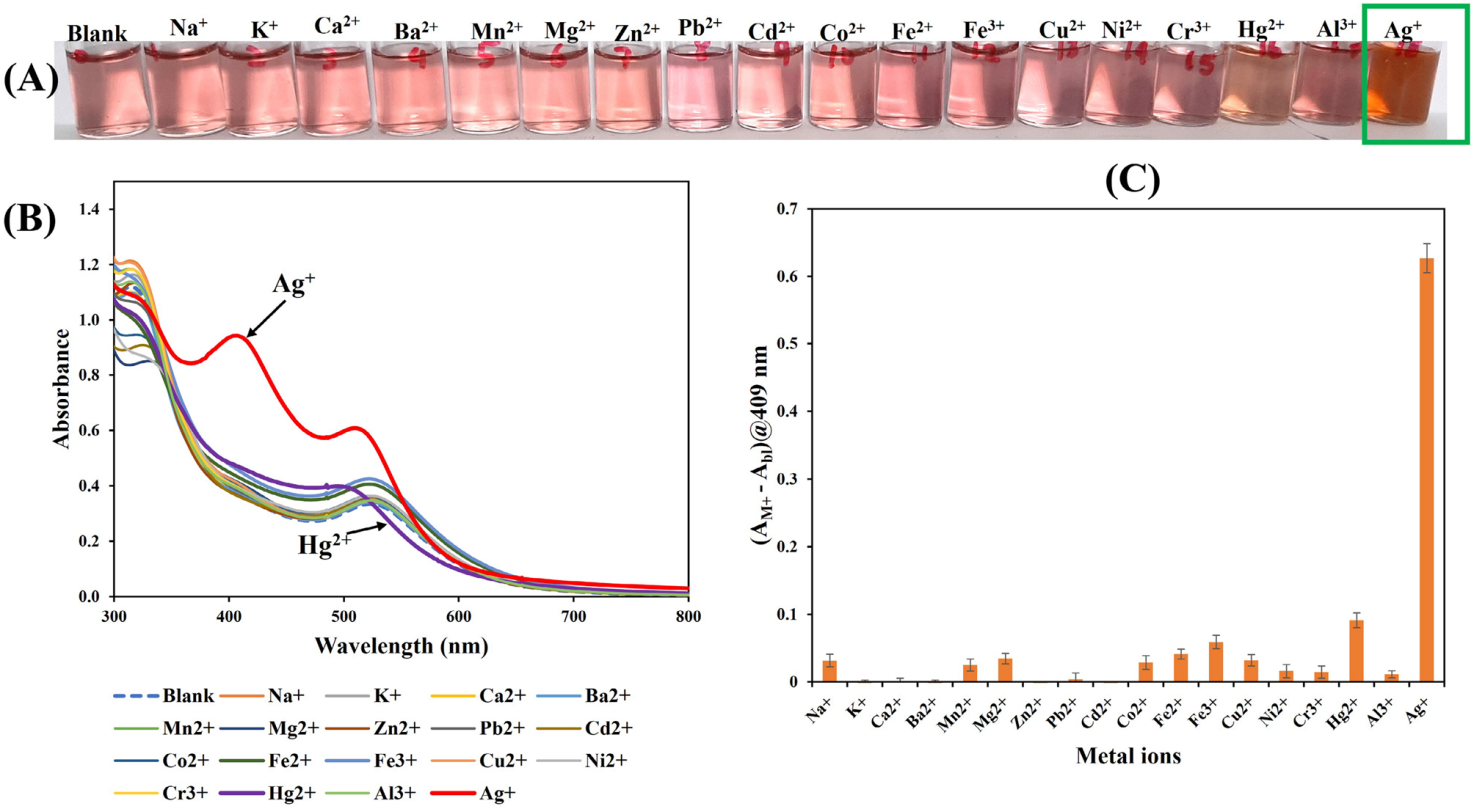
(**A**) Photo images, (**B**) UV–Vis absorption spectra of pH adjusted TA/PUL-AuNPs under different metal ion concentrations, Blank, Na^+^, K^+^, Ca^2+^, Ba^2+^, Mn^2+^, Mg^2+^, Zn^2+^, Pb^2+^, Cd^2+^, Co^2+^, Fe^2+^, Fe^3+^, Cu^2+^, Ni^2+^, Cr^3+^, Hg^2+^, Al^3+^ and Ag^+^, with Ag^+^  = 60.0 µM, while other metals are 120 µM. (**C**) Plot of (A_M+_ − A_bl_)@409 nm against different Metal ions.

The selectivity of the detection probe TA/PUL-AuNPs to respond to Ag^+^ in comparison with other numerous metal ions was investigated. The single response of Ag^+^ and other possible interfering metal ions (M^+^) in real environmental samples were charged on the TA/PUL-AuNPs probe and presented in Fig. 8. As revealed, the TA/PUL-AuNPs maintained its reddish color, in the presence of higher concentrations of other metals, except for Hg^2+^, where a slight faint color (Fawn color), was observed (Fig. 8A). The UV–Vis spectrum is shown in Fig. 8B, where absorption enhancement for Ag^+^ (Fig. 8B, red line) is clearly distinguishable from other metal ions. Hg^2+^ (Fig. 8B, purple line) on the other hand showed a blue shift, without a noticeable peak at 409 nm. The plot of response (A_M+_ − A_bl_)@409 nm against different metal ions (Fig. 8C), shows Ag^+^ response exceeding other metal ions. The response of the nearest metal ion (Hg^2+^) is calculated to be about 14% that of Ag^+^, even when Hg^2+^ was maintained at two-fold higher concentration. This shows that Hg^2+^ may induce slight interfering effect on Ag^+^ response. Hg^2+^ response is possible, since under the detection condition of Ag^+^, Hg^2+^ can form complex and equally be reduced by TA, to Hg^0^, which can be deposited on the AuNPs to yield Au@Hg amalgam^53^. To circumvent the effect of Hg^2+^, we investigated the use of EDTA as a masking agent, since it has a higher binding constant with Hg^2+^ than with Ag^+^^54^ and thus can be used to nullify Hg^2+^ slight response. The concentration of EDTA used was maintained at 1.25 mM, by adding 100 µL of 25 mM EDTA aqueous solution to the detection probe maintained at 2 mL reaction volume. Figure S12, shows the UV–Vis spectra of blank sample (pH adjusted TA/PUL-AuNPs) (Fig. S12, curve a), with red color (Fig. S12, image a-inset). The addition of EDTA to blank is shown in (Fig. S12, curve b-showing a diminished spectra intensity, with a fainter red color (Fig. S12, image b-inset). Hg^2+^ injection on blank, without EDTA is shown in (Fig. S12, curve c), showing Hg^2+^ alteration on the UV spectrum, with obvious observable color change (Fig. S12, image c-inset). Ag^+^ injection on blank is shown in (Fig. S12, curve d), revealing Ag^+^ mediated spectra enhancement with yellowish color (Fig. S12, image d-inset). EDTA effect on Hg^2+^ addition to blank is shown in (Fig. S12, curve e). Accordingly, the spectra shift of Hg^2+^ was nullified (almost like blank), validating the chelating capacity of EDTA to Hg^2+^. The color perturbation of Hg^2+^ on TA/PUL-AuNPs could be seen to be prevented with red color observed. However, with EDTA added to Ag^+^ and blank (Fig. S12, curve f), the UV spectra still shifted significantly, with obvious peak at 409 nm, while the photo image shows yellow coloration (Fig. S12, image f-inset). This observation validated that the slight Hg^2+^ response to the TA/PUL-AuNPs probe even at two-fold higher concentration of Ag^+^ can be prevented with the use of EDTA, without imparting on Ag^+^ response, hence this probe can be highly selective to Ag^+^ over other possible metal ions.

### Comparison with reported methods

The detection strength of the TA/PUL-AuNPs probe was compared to numerous reported methods for Ag^+^ detection, as presented in Table 1. Compared with methods based on electrochemical workstations using DNA as recognition molecules^9,10^, the present TA/PUL-AuNPs is cost effective, as bio-recognition molecules are generally very expensive. Besides, DNA is labile and is easily degraded, thus presenting challenges to assay´s robustness. Compared with the fluorometric assays^11,12^, the proposed TA/PUL-AuNPs probe shows, similar LOD and linear range, however, from the read-out devices, the TA/PUL-AuNPs based on the use of UV–Vis spectrophotometer is more affordable than the simplest fluorimeter. Thus, it can easily be used in economically challenging settings. In comparison to other reported colorimetric methods^9,13,15,55^, the proposed TA/PUL-AuNPs shows remarkable advantages, in terms of presenting a one-pot synthesis strategy without any need for further surface functionalization^15^. Moreover, while most of the detection methods relied mostly on AuNPs aggregation from (red to blue color), the color transition from red to yellow in this work, is in itself remarkable and thus adds to the strength of this detection strategy. It is relevant to posit that, while some of the reported probes were carried out using highly toxic materials. In terms of environmental sustainability, the TA/PUL-AuNPs are far better, by virtue of the materials used in the probe fabrication (TA and PUL). Thus, the present assay fits within the concept of “green chemistry”, benefiting from using non-toxic starting materials for nanoprobe fabrication. It is important to state that the color transitions in the TA/PUL-AuNPs towards Ag^+^ detection is completely different from the other AuNPs based nanoprobes, where color changes from red to blue or vice versa is often used to quantify the detection responses. As a result, the TA/PUL-AuNPs presents an impressive naked-eye based detection strategy, that would enrich literature especially in the design of optical detection assays for HMs monitoring in environmental samples.

**Table 1:** Comparison of TA/PUL-AuNPs probe with reported methods for Ag^+^ detection.

Detection methods	Linear range (µM)	Limit of detection, LOD (nM)	References
Electrochemistry (SWV)	5.00 × 10^–4^ − 0.100	0.100	^9^
Electrochemistry (DPV)	1.00 × 10^–4^ − 0.120	0.0300	^10^
Fluorimetry (N, S-Cdots)	0.100–700	30.0	^11^
Fluorimetry (N-CDs)	0.700–36.0	59.0	^12^
Colorimetry (g-C_3_N_4_-PtNPs)	5.00 × 10^–5^ − 5.00 × 10^–3^	0.022	^55^
Colorimetry (Thiamazole-AuNPs)	1.00 × 10^–4^ − 9.00	0.0420	^14^
Colorimetry (Hyperbranched polymer)	8.70 × 10^–3^ − 127	8.76	^56^
Colorimetry (Benzo-5-crown-AuNPs)	0.0200–0.950	12.5	^15^
Colorimetry (TRIS-AuNPs)	1.00 − 9.00	410	^13^
Colorimetry (TA/PUL-AuNPs)	0.100–150	30.8	This work

SWV = Square Wave Voltammetry; DPV = Differential Pulse Voltammetry; N, S, Cdots = Nitrogen, Sulphur doped carbon quantum dots; N-CDs = *Lycium ruthenicum* based Nitrogen carbon dots, g-C_3_N_4_-PtNPs = graphitic carbon nitride (g-C_3_N_4_) and platinum nanoparticles (PtNPs); TRIS = tris(hydroxymethyl) aminomethane.

### Application of TA/PUL-AuNPs for Ag^+^ detection in environmental sample

**Figure 9:**
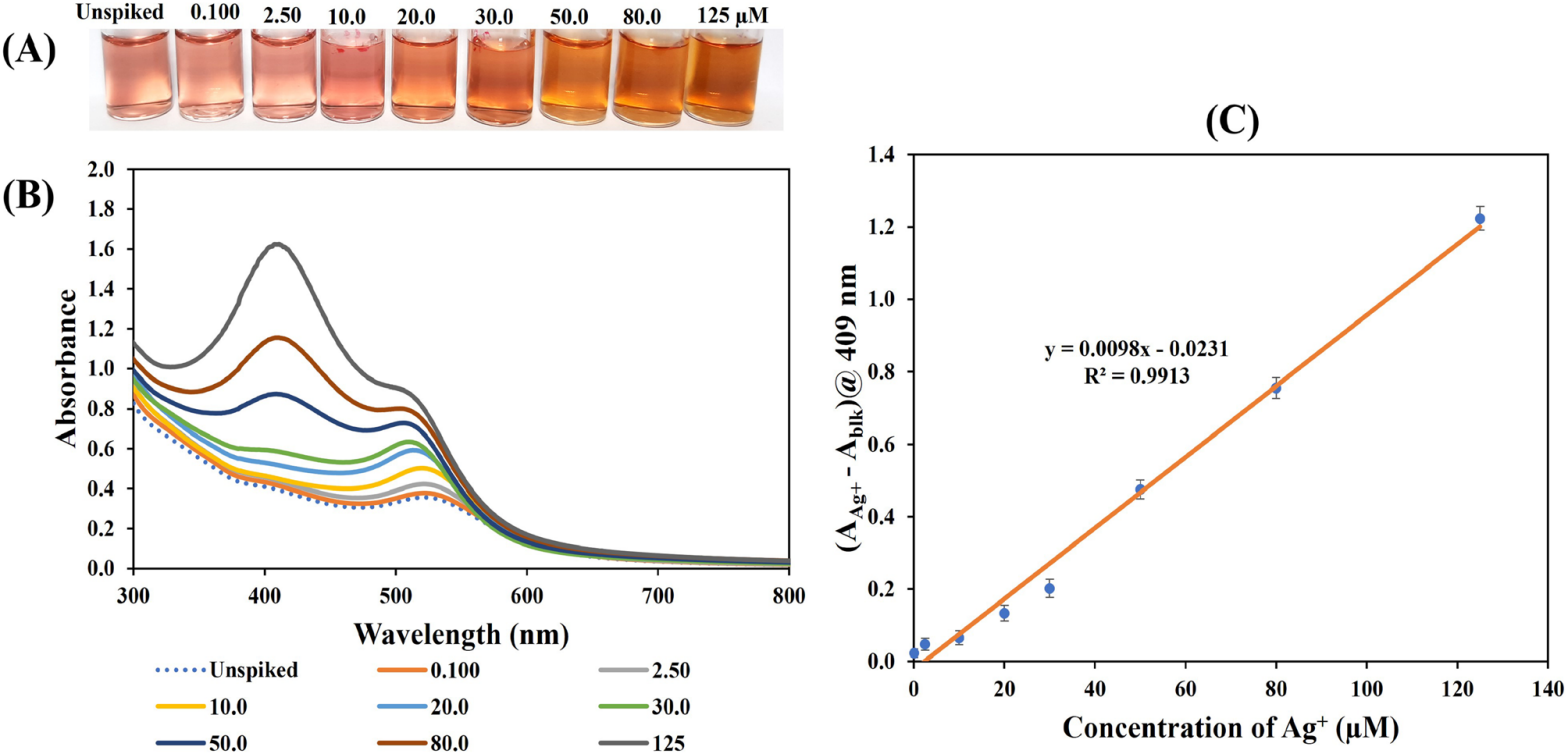
(**A**) Photo images, (**B**) UV–Vis spectra, of pH adjusted TA/PUL-AuNPs of unspiked and spiked Ag^+^ concentrations within (0.100–150 µM) in lake water sample. (**C**) Plot of (A_Ag+_ − A_bl_)@409 nm against Ag^+^ concentrations within linear range (0.100–150 µM).

To verify the detection efficacy of the TA/PUL-AuNPs probe for Ag^+^, in real environmental sample, we used lake water sample from the Chulalongkorn University, Thailand. The water sample was collected and treated as detailed in section 2.5. Ag concentration in the sample was profiled using ICP-OES and was not found in the sample. The standard addition method was applied for Ag^+^ detection using TA/PUL-AuNPs probe. Different Ag^+^ concentrations were spiked into the processed lake water sample and were charged on the probe, following the detection condition used for the calibration plot. The unspiked sample and Ag^+^ concentrations within (0.100–150 µM) i.e., the linear range from the calibration plot is shown in Fig. 9. Eight points were used, and the photo image is revealed in Fig. 9A. Accordingly, naked-eye color transitions from red, to orange and yellow were observed, just like the response from the calibration plot. This shows that the TA/PUL-AuNPs can respond to Ag^+^ detection in complex environmental samples. Figure 9B shows the UV–Vis absorption spectra, where hypsochromic peak shift was observed, while strong peak at 409 nm, characteristic of AgNPs is conspicuously revealed. The plot of (A_Ag+_ − A_bl_)@409 nm against Ag^+^ concentrations, spiked in lake water sample is shown in Fig. 9C. The plot generated an equation, (A_Ag+ _− A_bl_)@409 nm = 0.0098[Ag^+^] − 0.0231, with R^2^ value of 0.9913. The response in lake water sample and the calibration plot are compared to gain insight into lake sample matrix effect on the TA/PUL-AuNPs detection potentiality. As revealed in Fig. S13, both plots (calibration plot or Cal plot and Lake sample), when compared using a two-way ANOVA, shows there existed no significant differences between the two plots (P ≥ 0.05). Thus, the accuracy of the TA/PUL-AuNPs was estimated using the lake water sample plot. The recovery obtained are within 98.8 to 102.2% (Table S2), which shows the detection probe can reliably detect Ag^+^ in solution.

## Conclusion

This work proposed an environmentally friendly approach towards the synthesis of AuNPs, through synergistic combination of TA and biopolymer (Pullulan), to generate TA/PUL-AuNPs. TA was used as the reducing agent of gold salt, inside hydrophilic PUL polymer matrix. At the optimal synthesis condition, TA/PUL-AuNPs showed UV–Vis maximum at 529 nm. Detailed instrumental characterization was applied to obtain information on the optical, morphological, and microstructural properties of TA/PUL-AuNPs. Results showed that TA reduced gold salt to its nanoparticles, all inside PUL matrix. It was further shown that, while TA mediated synthesis generated highly stable and dispersed AuNPs, within PUL matrix, excess TA could be held, through intermolecular hydrogen bonding between TA and PUL. Consequently, after pH adjustment to 11, TA/PUL-AuNPs could be activated for Ag^+^ detection, through *in-situ* reduction of added Ag^+^ to AgNPs.  The formed AgNPs can be further deposited on the already-formed PUL-AuNPs to yield PUL-Au@Ag core shell nanostructure,  with observable color perturbations from red to orange and yellow. The detection mechanism was investigated in detail, and the optimal detection conditions, furnished a linear relationship between (A_Ag+ _− A_bl_)@409 nm  and Ag^+^ concentrations within 0.100–150 µM, while the calculated LOD was 30.8 nM. The TA/PUL-AuNPs was used for Ag^+^ detection in lake water samples with reliable accuracy.

## Supplementary Information

Supplementary Information.

## Data Availability

All data generated and analyzed for this work have been used in the major article and its supplementary information files.

## Abbreviations

TA: Tannic acid
AuNPsL: Gold nanoparticles
PUL: Pullulan
TA/PUL-AuNPs: Tannic acid/pullulan-gold nanoparticles
AgNPs: Silver nanoparticles
RT: Room temperature
EDTA: Ethylenediaminetetraacetic acid
NaOH: Sodium hydroxide
MNPs: Metal nanoparticles
XPS: X-ray photoelectron spectroscopy
