# Supplementary material for: Toxic Ag+ detection based on Au@Ag core shell nanostructure formation using Tannic acid assisted synthesis of Pullulan stabilized gold nanoparticles
Source: Sci Rep. 2023 Feb 1;13:1844. doi: 10.1038/s41598-023-27406-9 (PMC9892037; doi:10.1038/s41598-023-27406-9)
Supplement: Supplementary file 1 — Supplementary Information. [file 41598_2023_27406_MOESM1_ESM.docx]

**Supplementary Information**

**Toxic Ag^+^ detection based on Au@Ag core shell nanostructure formation using Tannic acid assisted synthesis of Pullulan stabilized gold nanoparticles**

**Titilope John Jayeoye^1^*, Chamaiporn Supachettapun^2^, & Nongnuj Muangsin^1,3^***

*^1^ Department of Chemistry, Faculty of Science, Chulalongkorn University, Bangkok 10330, Thailand*

*^2^ Program of Petrochemistry and polymer science, Faculty of Science, Chulalongkorn University, Bangkok 10330, Thailand*

*^3^ Nanotec-CU Center of Excellence on Food and Agriculture, Department of Chemistry, Faculty of Science, Chulalongkorn University, Bangkok 10330, Thailand*

**Corresponding authors,**

- N. Muangsin ([nongnuj.j@chula.ac.th](mailto:nongnuj.j@chula.ac.th); nongnuj.ms@gmail.com)
- T.J. Jayeoye (titilope12@gmail.com & jayeoye.t@chula.ac.th)

**Fig. S1** UV-vis absorption spectra of TA/PUL-AuNPs, showing the stability of the synthesized colloidal solution under the optimal condition of 2.5% PUL.


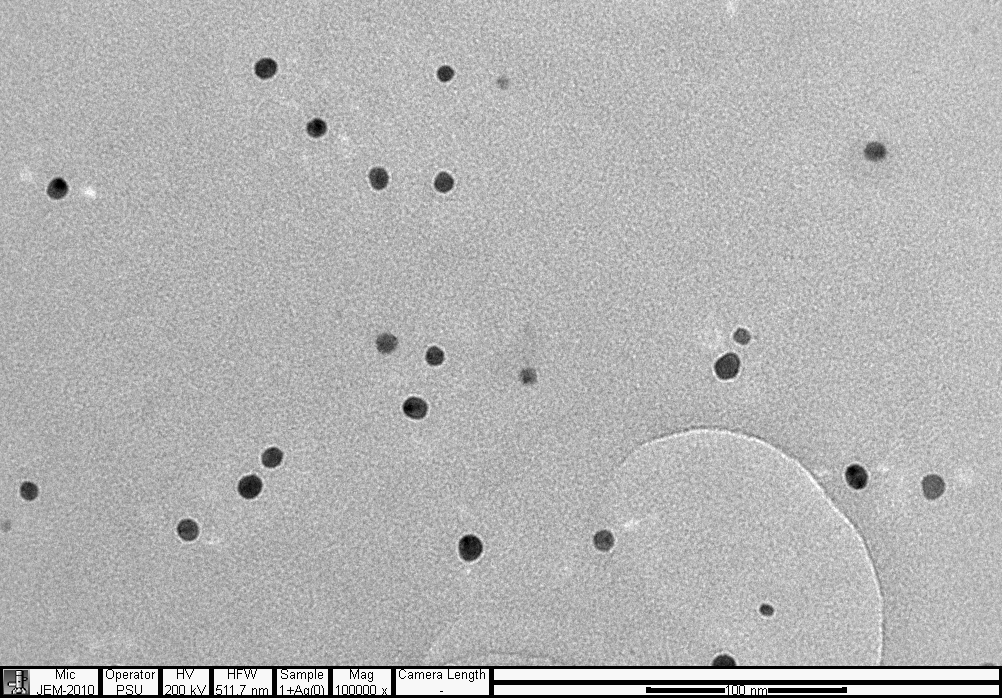


**100 nm**

**Fig. S2** TEM Images of TA/PUL-AuNPs

**Fig.S3** Plot of frequency against particle size of TA/PUL-AuNPs from Image J


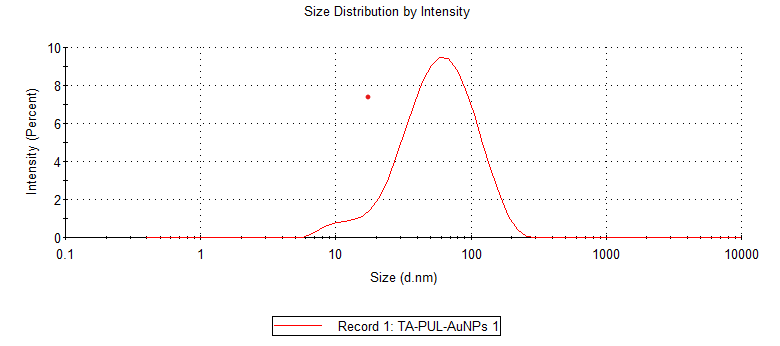


**D_h_ = 66.5 ± 1.7 nm**

**Fig.S4** Hydrodynamic size estimation of TA/PUL-AuNPs from DLS

**Zeta = -15.4 ± 0.8 mV**


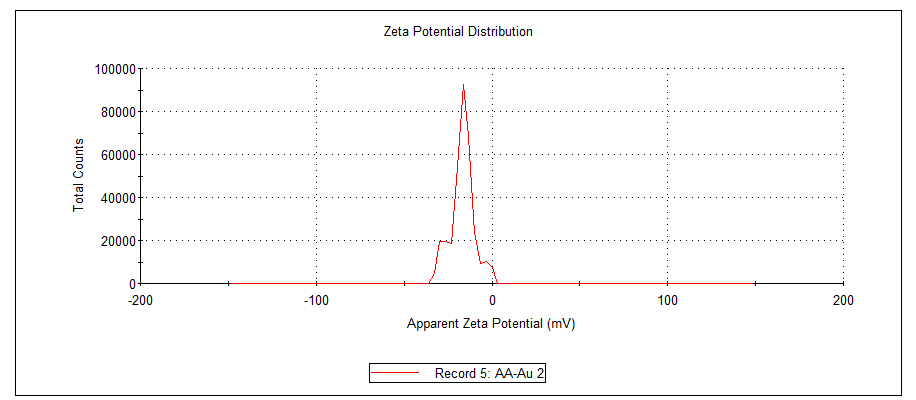


**Fig.S5** Zeta potential of TA/PUL-AuNPs

**
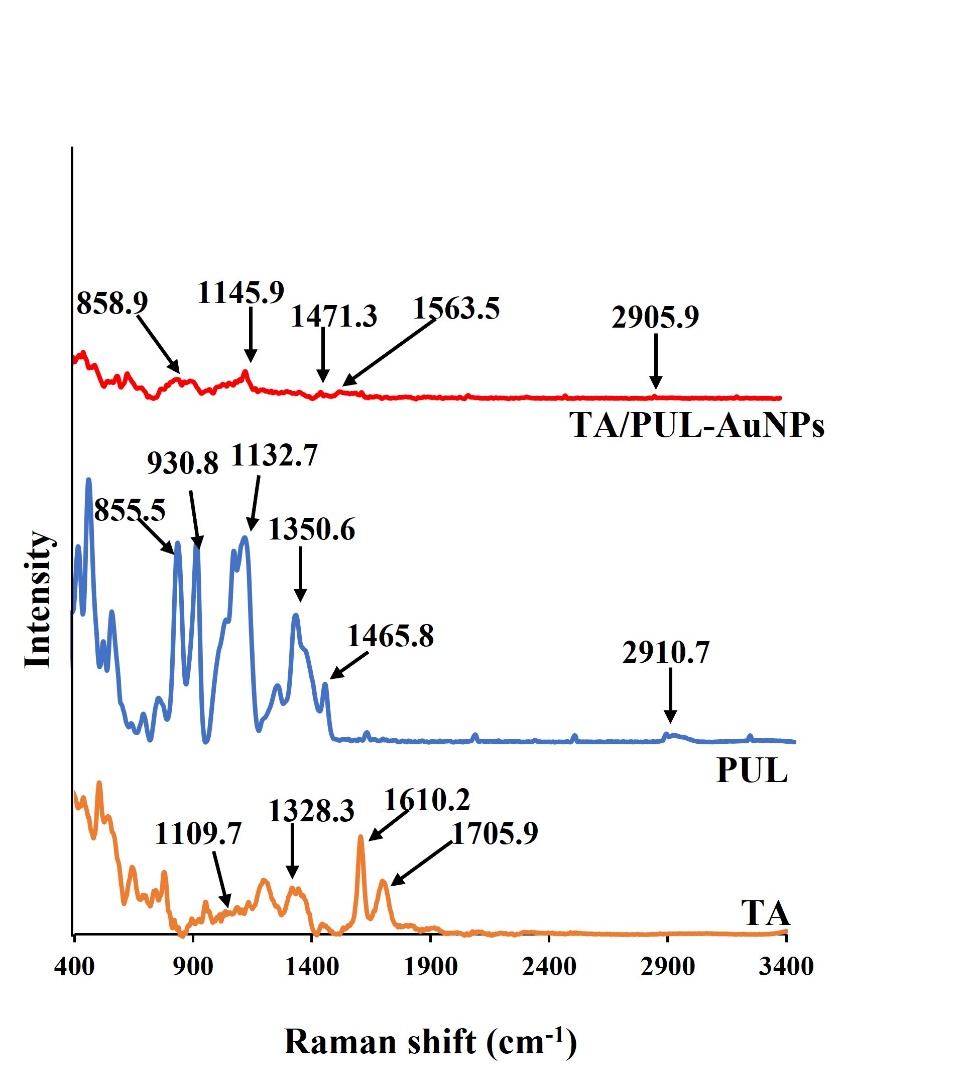
**

**Fig.S6** Raman spectra of TA, PUL and TA/PUL-AuNPs

**Table S1:** TGA of PUL and TA/PUL-AuNPs

| **Sample** | **Stages** | **Onset Temperature (°C)** | **Temperature ranges (°C)** | **IDT (°C)** | **IPDT (°C)** | **Mass loss (%)** | **Ash (%)** |
| --- | --- | --- | --- | --- | --- | --- | --- |
| PUL | I | 25.30 | 25.30-250.4 | 25.30 | 900.0 | Y = 6.7 | 3.50 |
|  | II | 250.4-500.5 | 250.5-500.1 |  |  | Y = 78.1 |  |
|  | III | 500.1 | 500.1-900.0 |  |  | Y = 11.7 |  |
|  |  |  |  |  |  | Total = 96.5 |  |
| TA/PUL-AuNPs | I | 25.40 | 25.4-250.4 | 51.50 | 900.0 | Y = 4.1 + 13.8 | 22.1 |
|  | II | 250.4 | 250.4-900.0 |  |  | Y = 60.0 |  |
|  |  |  |  |  |  | **Total = 77.9** |  |

**IDT: Initial decomposition temperature; IPDT: Integral procedural decomposition temperature.**


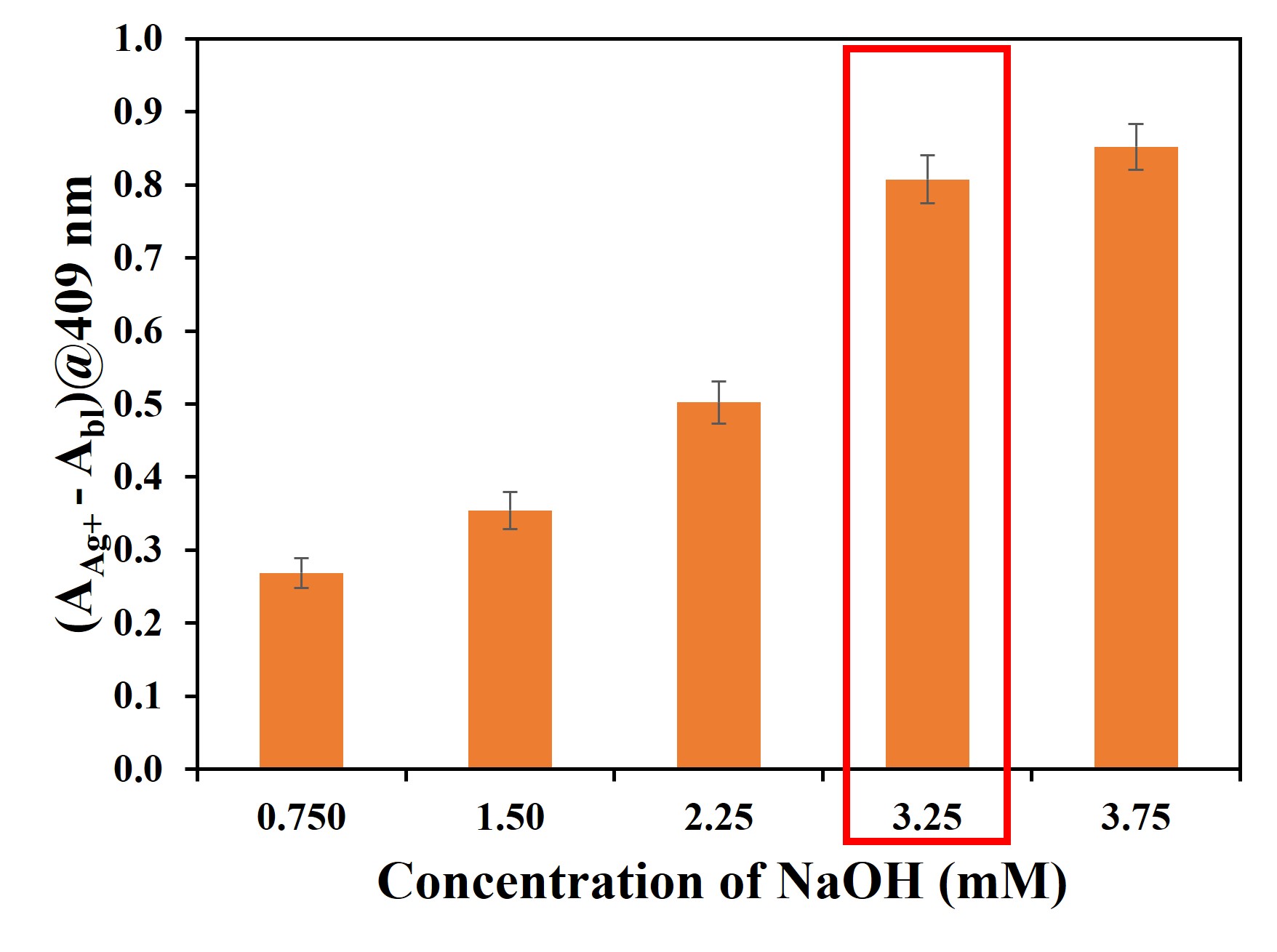


**Fig. S7** Plot of NaOH concentration against response, under 80.0 µM Ag^+^


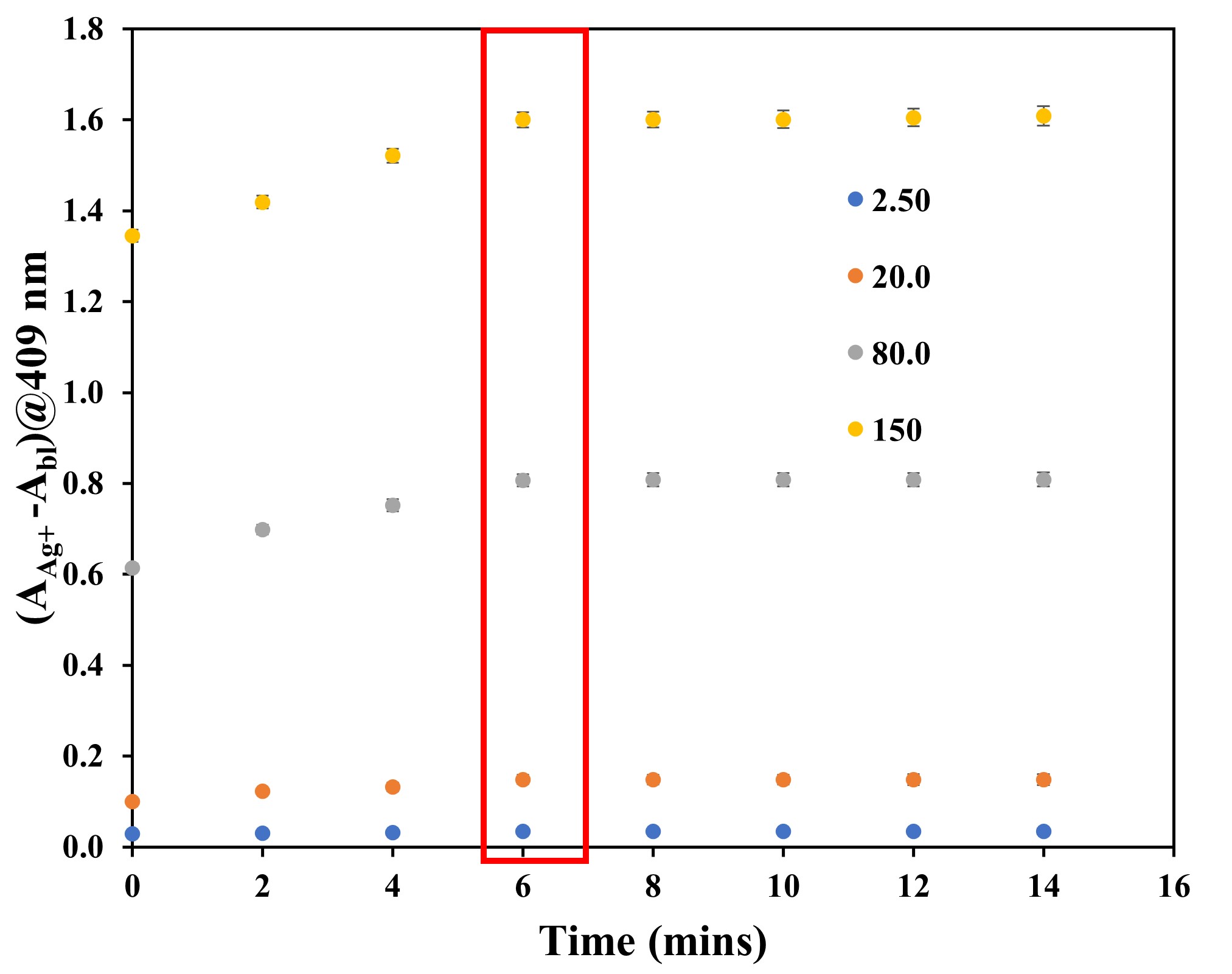


**Fig. S8** Plot of incubation Time (min) against response, under different concentrations of Ag^+^

(2.50, 20.0, 80.0 and 150 µM)

**Fig. S9** UV-vis absorption spectra of TA/PUL-AuNPs showing Ag^+^ concentrations from 0.000 to 5.00 µM.

**Fig.S10** Zeta potential of pH adjusted TA/PUL-AuNPs under Ag^+^ injection at 0.000 (Blank), 30.0 and 100 µM.


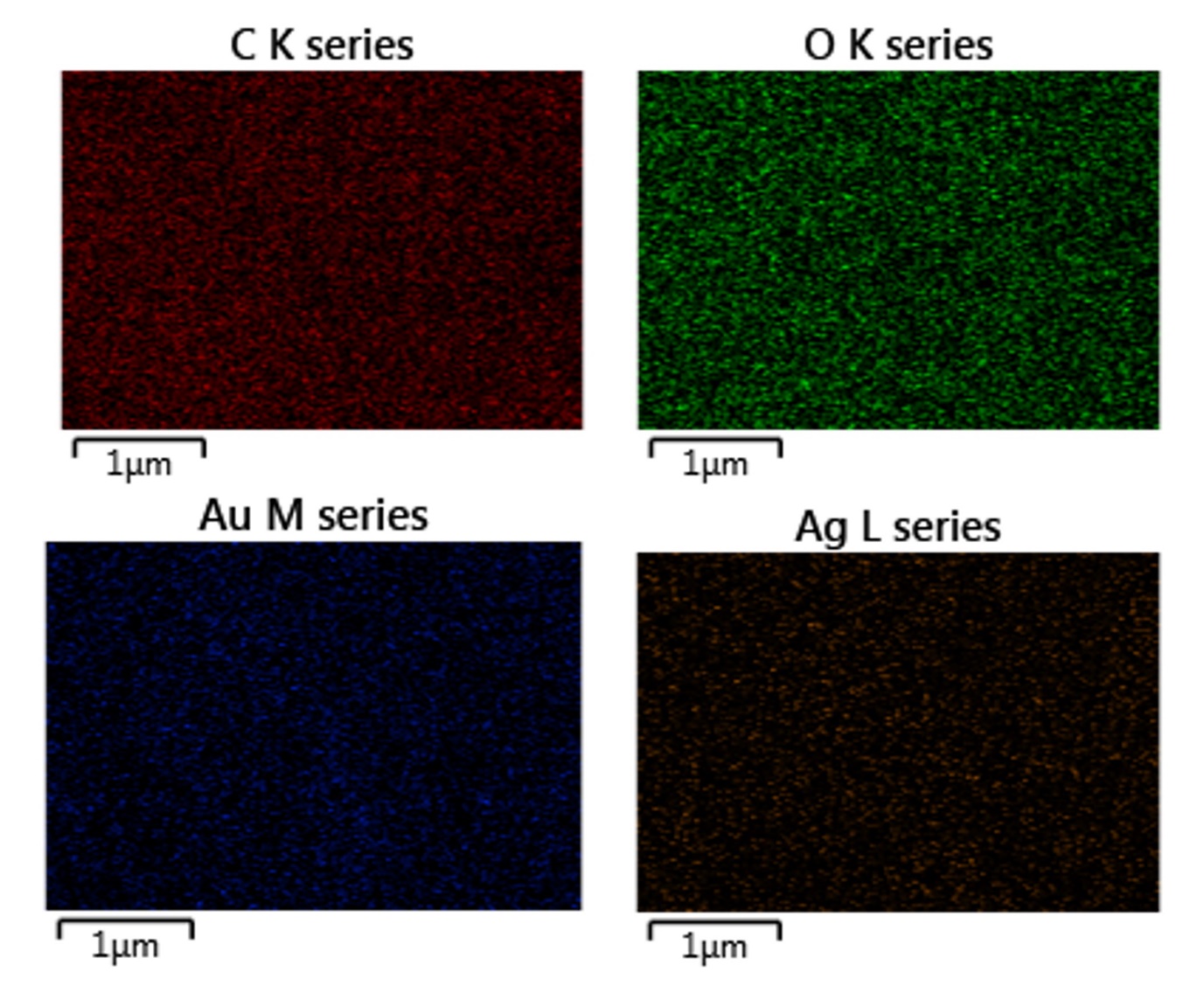


**Fig. S11** Elemental mapping of TA/PUL-AuNPs, revealing C, O Au and Ag.


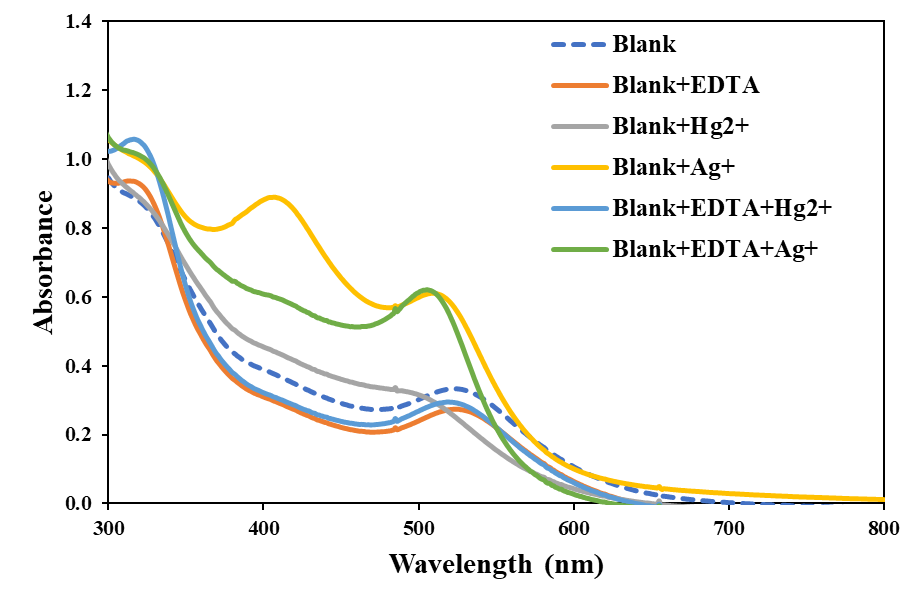

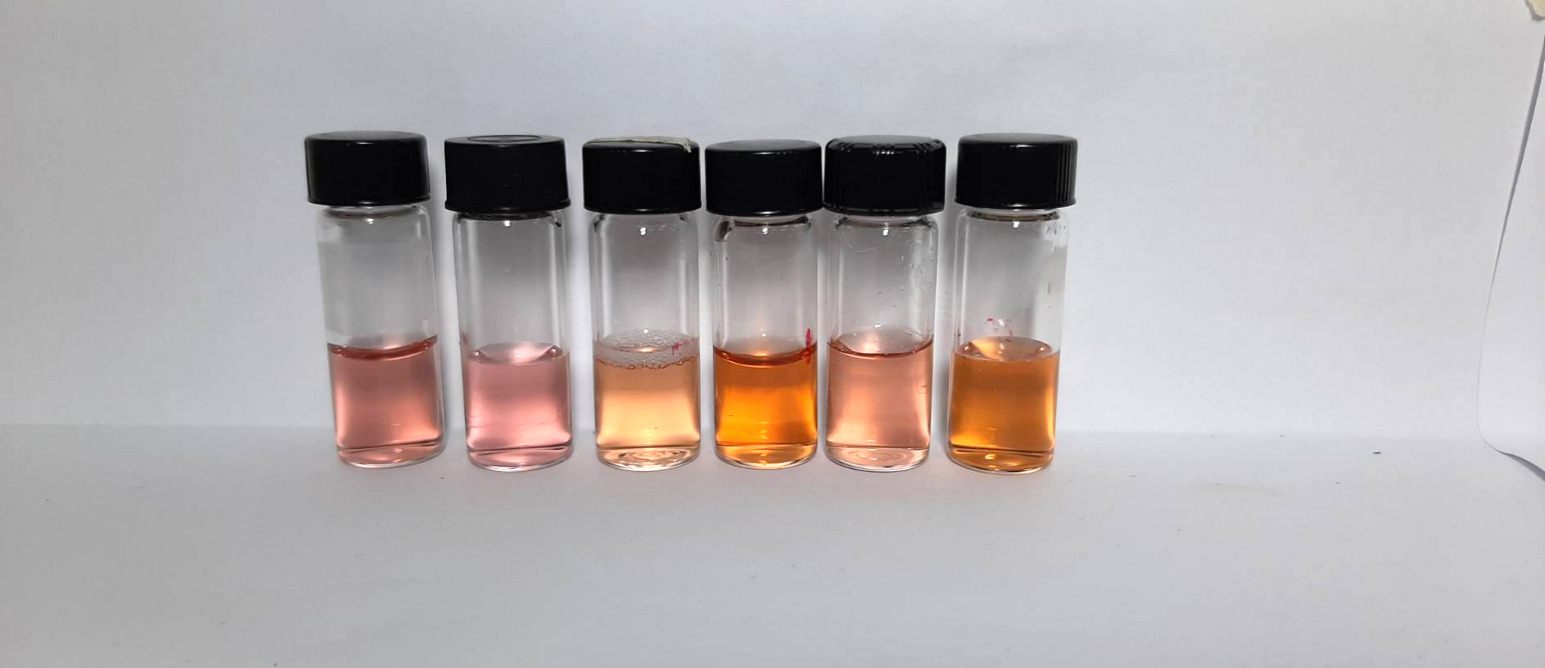


**a**

**f**

**e**

**d**

**b**

**c**

**a**

**f**

**e**

**d**

**c**

**b**

**Fig. S12** UV-vis absorption spectra of pH Adjusted TA/PUL-AuNPs optical response in the presence of EDTA as a masking agent for Hg^2+^ and Ag^+^, with [Ag^+^] = 60.0 µM and [Hg^2+^] = 120 µM, [EDTA] = 1.25 mM.

**Fig. S13** Plot of (A_Ag+_ - A_bl_)@409 nm against Ag^+^ concentrations (calibration plot and Lake sample) within linear range (0.100-125 µM).

**Table S2** Determination of Ag^+^ in lake water sample based on TA/PUL-AuNPs

| **Concentration added (**µM**)** | **Concentration found (**µM**)** | **Recovery (%)** | **RSD (%)**  **n = 3** |
| --- | --- | --- | --- |
| 0.0 | 0.00 | - | - |
| 2.50 | 2.47 ± 0.15 | 98.80 | 1.4 |
| 20.0 | 20.30 ± 0.11 | 101.5 | 1.9 |
| 50.0 | 51.10 ± 0.22 | 102.2 | 2.8 |
